# A novel approach for quantitative electrogram analysis for driver identification: Implications for ablation in persistent atrial fibrillation

**DOI:** 10.3389/fcvm.2022.1049854

**Published:** 2022-12-01

**Authors:** Wen-Rui Shi, Shao-Hui Wu, Guang-Chen Zou, Kai Xu, Wei-Feng Jiang, Yu Zhang, Mu Qin, Xu Liu

**Affiliations:** ^1^Department of Cardiology, Shanghai Chest Hospital, Shanghai Jiao Tong University, Shanghai, China; ^2^Department of Internal Medicine, Danbury Hospital, Danbury, CT, United States

**Keywords:** persistent atrial fibrillation, catheter ablation, AF driver mapping, multiscale entropy, long-term outcomes

## Abstract

**Objective:**

This study sought to study the feasibility, efficacy, and safety of using multiscale entropy (MSE) analysis to guide catheter ablation for persistent atrial fibrillation (PsAF) and predict ablation outcomes.

**Methods:**

We prospectively enrolled 108 patients undergoing initial ablation for PsAF. MSE was calculated based on bipolar intracardiac electrograms (iEGMs) to measure the dynamical complexity of biological signals. The iEGMs data were exported after pulmonary vein isolation (PVI), then calculated in a customed platform, and finally re-annotated into the CARTO system. After PVI, regions of the highest mean MSE (mMSE) values were ablated in descending order until AF termination, or three areas had been ablated.

**Results:**

Baseline characteristics were evenly distributed between the AF termination (*n* = 38, 35.19%) and the non-termination group. The RA-to-LA mean MSE (mMSE) gradient demonstrated a positive gradient in the non-termination group and a negative gradient in the termination group (0.105 ± 0.180 vs. −0.235 ± 0.256, *P* < 0.001). During a 12-month follow-up, 29 patients (26.9%) had arrhythmia recurrence after single ablation, and 18 of them had AF (62.1%). The termination group had lower rates of arrhythmia recurrence (15.79 vs. 32.86%, Log-Rank *P* = 0.053) and AF recurrence (10.53 vs. 20%, Log-Rank *P* = 0.173) after single ablation and a lower rate of arrhythmia recurrence (7.89 vs. 27.14%, Log-Rank *P* = 0.018) after repeated ablation. Correspondingly, subjects with negative RA-to-LA mMSE gradient had lower incidences of arrhythmia (16.67 vs. 35%, Log-Rank *P* = 0.028) and AF (16.67 vs. 35%, Log-Rank *P* = 0.032) recurrence after single ablation and arrhythmia recurrence after repeated ablation (12.5 vs. 26.67%, Log-Rank *P* = 0.062). Marginal peri-procedural safety outcomes were observed.

**Conclusion:**

MSE analysis-guided driver ablation in addition to PVI for PsAF could be feasible, efficient, and safe. An RA < LA mMSE gradient before ablation could predict freedom from arrhythmia. The RA-LA MSE gradient could be useful for guiding ablation strategy selection.

## Introduction

Atrial fibrillation (AF) is the most common cardiac arrhythmia and contributes to morbidity and mortality (1). At its cornerstone, catheter ablation with pulmonary vein (PV) isolation (PVI) has been established as a treatment option for paroxysmal and persistent symptomatic AF. However, rhythm outcome is far from assured for persistent AF (PsAF) ablation, with reported freedom from AF of only 20–30% 1-year post-procedure (2).

Identifying and targeting mechanisms for AF maintenance or AF drivers is likely the key to improving the efficacy of AF ablations. Multiple mechanisms have been proposed to initiate and maintain AF; a localized driver caused by the rotor is one of the major mechanisms (3, 4). Early basic studies have identified that stable, self-sustained rotors can exist in the atria. High-frequency activation by such rotors results in the complex patterns of activation that characterize AF (5). Lately, by optically mapping diseased human right atria *ex vivo*, Hansen et al. revealed that the complex atrial microstructure caused significant differences between Endo vs. Epi activation during pacing and sustained AF driven by intramural re-entry anchored to fibrosis-insulated atrial bundles (6). Most importantly, the results from CONFIRM trial (Conventional Ablation for Atrial Fibrillation With or Without Focal Impulse and Rotor Modulation) have demonstrated the superiority of focal impulse and rotor modulation plus conventional ablation over conventional ablation alone regarding the acute AF termination rate and long-term freedom from AF rate (7, 8). However, the CONFIRM trial used a specialized basket mapping catheter to acquire a computational AF map, but the catheter is sometimes unavailable in the current clinical practice. Therefore, there is a demand to develop algorithms based on existing hardware conditions to improve the detection of the core of rotors.

Many mapping algorithms have been developed to guide driver-based AF ablation, such as local activation time, complex fractionated electrograms, and dominant frequency (DF) mapping (9–11). While incremental benefit has been reported with some of these approaches, other studies have shown highly frustrating results. This phenomenon is likely in part due to the technical limitations of the methods, which led to inaccurate identification of drivers (12). These approaches are still far from achieving further technological advances to decrease the gap between the gold-standard optical mapping and current clinically available technology.

The multiscale entropy (MSE) technique was recently proposed for coarse-grained time-scaling procedures to offer a more robust determination of the complexity of time series data (13). MSE is a quantitative method developed to measure the dynamical complexity of biological signals on multiple spatial and temporal scales. Although MSE has been used to accurately identify rotor cores using optical mapping data from rabbit hearts and computer-simulated human intracardiac electrograms (iEGMs) (14–16), the feasibility, efficacy, and safety of MSE-guided driver ablation in the clinical practice are still not clear. Accordingly, our study aimed to evaluate the feasibility, efficacy, and safety of using the mean MSE (mMSE)-based AF driver mapping technique to guide ablation and predict rhythm outcomes in patients with persistent atrial fibrillation (PsAF).

## Materials and methods

The study protocol was approved by the Institutional Review Board/Ethics Committee of Shanghai Chest Hospital (KSY21312). Written informed consent was obtained from every participant.

### Study population

**FIGURE 1:**
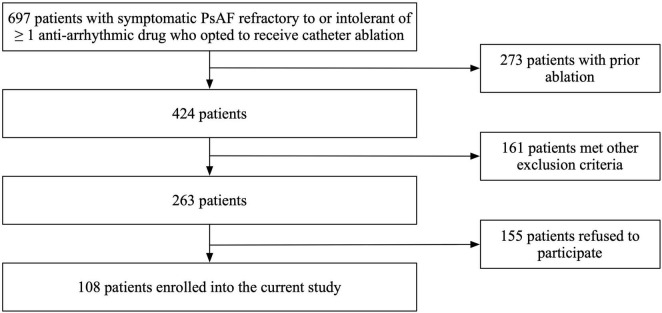
Flow chart of the enrollment process.

In this prospective cohort study, patients with symptomatic PsAF refractory to or intolerant of at least one antiarrhythmic drug who opted to receive catheter ablation were included. Key exclusion criteria included prior left atrial ablation (surgical or catheter), valvular heart disease, cardiomyopathy, severe pulmonary disease, presence of intracardiac thrombi, cardiac surgery, renal failure, thyroid dysfunction, and a left ventricular ejection fraction (LVEF) < 35% or left atrial diameter of ≥60 mm measured on transthoracic or transesophageal echocardiogram. A total of 697 patients with persistent AF were screened in our study. Among them, 273 subjects were excluded due to prior ablation; 161 subjects were excluded due to valvular heart disease, cardiomyopathy, severe pulmonary disease, presence of intracardiac thrombi, cardiac surgery, renal failure, thyroid dysfunction, and a left ventricular ejection fraction (LVEF) < 35% or left atrial diameter of ≥ 60 mm measured on transthoracic or transesophageal echocardiogram; and 155 subjects were excluded because they refused to participate in our study (Figure 1). Finally, we enrolled 108 consecutive patients undergoing their first RFA procedure for PsAF from November 2019 to December 2020 and completed a 12-month follow-up. All variables in this study, including baseline characteristics and echocardiographic parameters, were prospectively collected.

### Electrophysiological study

The electrophysiology study was performed after discontinuing antiarrhythmic medications for five half-lives or more than 60 days for amiodarone. Catheters were advanced from the femoral veins to the right atrium (RA), coronary sinus (CS), and transeptally to the left atrium (LA). A decapolar mapping catheter (Biosense Webster, Inc., Diamond Bar, CA, USA) was positioned in the CS *via* the left femoral vein. Two SL1-type Swartz sheaths (St. Jude Medical, St. Paul, MN, USA) were advanced into the LA after two successful transseptal punctures. After transseptal catheterization, systemic anticoagulation was achieved with intravenous heparin (100 IU/kg) to maintain an activated clotting time between 300 and 350 s. Selective PV venography was performed to identify all PV ostia before ablation. PentaRay NAV catheter (Biosense Webster, Inc., Diamond Bar, CA, USA) was used as a navigational mapping catheter.

### Multiscale entropy analysis and driver mapping

**FIGURE 2:**
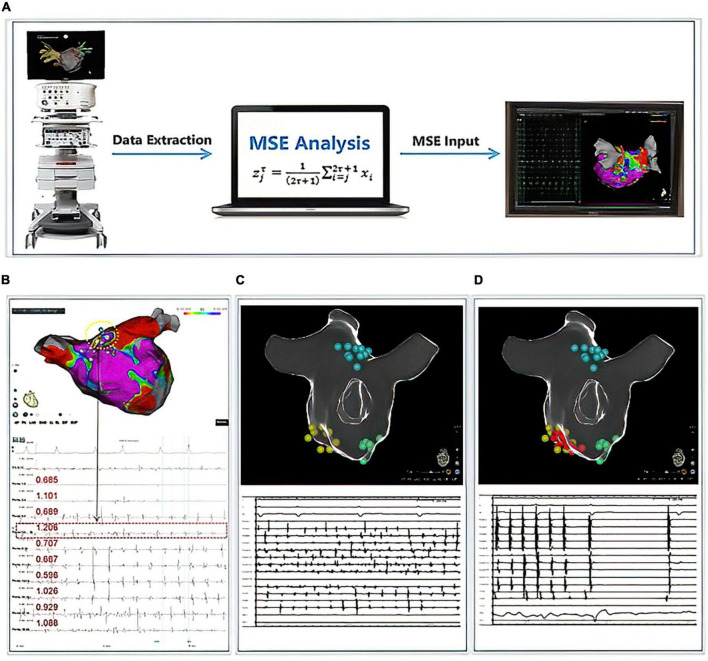
Multiscale entropy (MSE) calculation process and cases display. Panel **(A)** demonstrated the workflow for the calculation of MSE. Panel **(B)** displayed 1 case with successful AF termination by mMSE-guided ablation. The termination sites were located in the dotted circles, and the MSE values were listed below. Panels **(C,D)** displayed another case; yellow points indicated the area with the highest mMSE value, green points indicated the area with the second highest mMSE value, blue points indicated the area with the third highest mMSE value. We ablated the area with yellow points, and the AF terminated into sinus rhythm during the ablation. Therefore, we stopped the mMSE-guided ablation.

The MSE-based driver mapping was performed after PVI, and all MSE maps were performed under AF. Detailed data collection and MSE analysis approach can be founded in a prior research (16) and our Supplementary material. In brief, the MSE analysis approach could be divided into three major parts (displayed in Figure 2): (1) Mapping Data Collection: During an electrophysiological study, the iEGMs were acquired from both LA and RA. The CARTO (Biosense Webster, Inc., Diamond Bar, CA, USA) system was utilized to perform the electro-anatomical mapping. The high-resolution PentaRay NAV catheter was employed through a sequential scanning approach to map both atria evenly and thoroughly. Real-time 3D geometry of LA and RA were reconstructed. The CARTO system recorded both unipolar and bipolar iEGMs at a frequency of 1000 Hz, allowing the electrophysiological information to be coded with color and attached to the reconstructed model of LA and RA. An automated point collection mode (ConfiDENSE Continuous mapping) was used to avoid inappropriate point collection when electrodes did not contact the inner face of the atria. This mode allows point collection only when an appropriate contact is detected at the tip of the catheter. Multiple points were taken to create geometry with a fill threshold of 20 with uniform distribution across both chambers. (2) Data extraction and analysis: When the mapping was completed and the iEGMs data stored in the CARTO database, iEGMs data from both atria were extracted from the CARTO database manually and exported in the format of SAV. For MSE analysis, bipolar electrograms recorded by ten specified electrode pairs in the PentaRay catheter were needed for every mapping site. Due to the restriction of the CARTO system, a maximum of 2.5 s of electrograms at each mapping site were exported along with the surface ECG. The raw iEGMs were imported into a customized analyzing platform (Eclipse, solvusoft, Las Vegas, NV, USA). After that, the desired ten bipolar electrogram signals from the PentaRay catheter and surface ECG signals were captured with the assistance of artificial intelligence. After pre-processing noise canceling, the MSE values were calculated based on the ten bipolar electrograms and sorted automatically. In the current study, we used the mean MSE (mMSE) value of each mapping site rather than the MSE value of each electrode pair to construct the 3D MSE map due to technical limitations. Finally, the results were exported in the format of TXT. (3) Patient-specific 3D MSE map construction: The top five calculated mMSE values at each atrium were manually re-annotated back to the mapping sites from where the electrograms came from. The annotated mMSE data were then superimposed on the anatomical map to obtain the patient-specific 3D MSE distribution in the atria by interpolating the CARTO points for visualization. The data from our pre-study exploration of the mMSE-guided driver ablation demonstrated that the mean of all MSE values in the left or right atrium did not associate with intraprocedural termination. However, the top 5 MSE values differed between groups. Therefore, to facilitate and shorten the calculation process of MSE analysis, our current analysis only used the mMSE value of the top five mapping sites (When calculating mMSEs, we rank the mMSE values in both LA and RA individually) in each atrium. The RA-to-LA mMSE gradient was calculated as the difference between the means of the five highest mMSE values from RA and LA (equation: RA-to-LA mMSE gradient = mean of the top five mMSE values in RA—mean of the top five mMSE values in LA).

### Ablation protocol

All patients presented with AF. mMSE-guided driver ablation plus circumferential PVI was performed in all patients. PVI was performed after the construction of the 3D atrial model. PVI was conducted *via* an Ablation index-guided circumferential approach during AF (17, 18). After PVI, driver mapping was performed in the LA and RA. High-density MSE mapping at each atrium was performed with > 600 points in each patient (at least 300 surface points for each atrium were requested) using a PentaRay catheter with an interspace of 3.0 mm.

The mMSE-guided driver ablation was only performed in LA under the guidance of the CARTO system in descending order according to the mMSE value. ThermoCool SmartTouch (STSF) Catheter (Biosense Webster, Inc., Diamond Bar, CA) was used to perform the ablation. Radiofrequency power output was 45 W, the temperature was 43°C, the ablation time for each lesion was 20–30 s, and the saline infusion rate was 20–25 ml/min. We attempted to homogenize all tissue in about 3 cm^2^ for each targeted mapping region. The septum was mapped from both RA and LA, and if the top five mMSE values were located at any side of the septum, we ablated the septum from the left side of the septum. We sought a complete “flattening” of the bipolar signal amplitude at each radiofrequency application location. The primary endpoint of ablation was AF termination, defined as conversion to SR or a stable atrial flutter/tachycardia (AFL/AT). If the AF was not terminated after the targeted mapping area ablation, cardioversions were delivered to restore SR. Dechanneling was done by linear ablation, including mitral isthmus ablation, roofline ablation, and anterior line ablation, if necessary, based on the substrates. Transduction block was verified for each linear ablation, we strived to achieve a transduction block, and there was a mandate for a block in the tricuspid and the roof line but no mandate for the mitral line if failed after multiple ablation approaches. When ablation of the driver regions resulted in rhythm regularization into AFL/AT, the AFL/AT was mapped and ablated. No more than three targeted mapping regions were ablated to prevent over-ablation for each patient. For repeat procedure, if recurrence of AT/AFL, mapping of the arrhythmia was conducted, and dechannelling was performed according to the mapping results, reinforcement of PVI was conducted according to the operator’s judgment; if recurrence of AF, PVI was re-conducted, and then the ablation strategy was the same as the first procedure.

### Follow-up

All patients were hospitalized for at least 3 days following the ablation procedure, and cardiac rhythm was continuously monitored during the first 48 h. Antiarrhythmic medications were discontinued in all patients 3 months after the ablation. Outpatient visits and 48 h Holter monitoring were scheduled at 1, 3, 6, 9, 12 months, and every 6 months if the patient remained asymptomatic. Monthly telephone interviews were also done. All patients were asked to undergo additional ECGs and 7-day Holter recordings when their symptoms suggested tachycardia. A “recurrence” of atrial arrhythmia was considered any episode lasting 30 s (symptomatic or asymptomatic) detected by electrocardiography and/or Holter 3 months after the initial ablation procedure.

### Study endpoints

The study’s primary endpoint was acute AF termination during the procedure (7, 19). Secondary endpoints included: (1) freedom from atrial arrhythmia (AF/AFL/AT) after a single procedure on and off antiarrhythmic medications (excluding the first 3 months after ablation) at 12 months; (2) freedom from AF/AFL/AT after repeated ablation procedures at 12 months; (3) incidence of peri-procedural complications.

### Statistical analysis

Continuous variables were expressed as mean ± standard deviation (SD) or median with interquartile range and compared using independent sample *t*-tests or non-parametric tests. In contrast, categorical variables were expressed as percentages and compared using Chi-square tests. Survival curves were performed using the Kaplan–Meier (KM) curve, and comparisons were performed using the Log-rank test. Cox proportional hazards regression model was used to identify the association between intraprocedural AF termination and freedom from arrhythmia and AF following ablation. All tests were two-sided, with a probability of <0.05 to be considered significant. All statistical analyses were performed using STATA (version 15.0, StataCorp, TX, USA) and SPSS (IBM, Armonk, NY, USA).

## Results

### Baseline characteristics

Baseline characteristics were summarized in Table 1. The AF termination rate was 35.19%. The results showed marginal differences in age, AF duration, medication, CHA2DS2-VASc score, HAS-BLED score, and LVEF between groups. Patients in the AF termination group had a higher male percentage and higher rates of hypertension, diabetes, structural heart disease, and stroke/transient ischemic attack (TIA) history than patients in the non-termination group, but these differences were statistically insignificant. Notably, the results displayed a trend toward a smaller LA anteroposterior diameter in the termination group (*P* = 0.052).

**TABLE 1:** Baseline characteristics of enrolled subjects.

	All patients (*n* = 108)	Termination (*n* = 38)	Non-termination (*n* = 70)	*P*-value*
Age (year)	64.27 ± 10.41	65.37 ± 10.37	64.28 ± 9.95	0.593
Male (%)	80 (71.43)	14 (36.84)	16 (23.19)	0.132
AF duration (m)	24 (6–60)	24 (6–63)	24 (7–54)	0.634
**Medication**				
Number of failed AADs	2 (1–2)	2 (1–2)	2 (1–2)	0.605
Amiodarone (%)	103 (91.96)	34 (89.47)	64 (81.01)	0.519
**Comorbidity**				
Hypertension (%)	60 (53.57)	23 (79.31)	34 (43.04)	0.264
Diabetes (%)	11 (9.82)	6 (20.69)	5 (6.33)	0.164
Structural heart disease (%)	8 (7.21)	4 (13.79)	4 (5.06)	0.385
Stroke/TIA (%)	10 (8.93)	5 (17.24)	4 (5.06)	0.189
CHA2DS2-VASc score	2.09 ± 1.36	2.32 ± 1.53	2 ± 1.25	0.251
HAS-BLED score	1.51 ± 1.08	1.68 ± 1.19	1.43 ± 1.02	0.257
LA anterio-posterior diameter (mm)	44.45 ± 5.44	42.97 ± 5.16	45.13 ± 5.58	0.052
LVEF (%)	61.63 ± 8.58	62.87 ± 4.90	60.69 ± 10.20	0.218

Data were summarized as mean (SD), median (quartile 1–quartile 3), and numbers (percentage) according to their data type and distribution.

*Student’s *t*-test or Mann–Whitney test was used to compare continuous variates between groups. Chi-square test and Rank-sum test were used to compare categorical variables between groups.

AADs, antiarrhythmic drugs; TIA, transient ischemic attack; LA, left atrium; LVEF, left ventricular ejection fraction.

### Acute procedure outcome and intraprocedural parameters

Among the patients, no patients presented with AF termination after PVI. 38 (35.19%) patients had AF termination during the mMSE-guided ablation procedure, while 70 patients converted to sinus rhythm (SR) through electrical cardioversion (Table 2). In the AF termination group, 17 (44.74%) patients converted to SR, while 21 (55.26%) patients converted to AFL/AT. The mean value of the LA mapping point number and the RA mapping point number were not significantly different between the non-termination and termination groups (609.93 ± 35.77 vs. 615.72 ± 32.73, *P* = 0.409 and 356.80 ± 31.72 vs. 349.04 ± 26.02, *P* = 0.199, respectively). As measured by the CARTO system, the non-termination group had a significantly larger LA volume than the termination group (141.04 ± 31.54 ml vs. 123.13 ± 28.35 ml, *P* = 0.004). However, in the termination group, patients who converted to SR had smaller LA volumes than patients who converted to AT/AFL (116.47 ± 26.60 ml vs. 128.52 ± 29.20 ml, *P* = 0.196). Similarly, the results revealed that RA volume was significantly larger in the non-termination group than in the termination group (69.84 ± 18.41 ml vs. 60.42 ± 15.93 ml, *P* = 0.009), but the subgroups in the termination group did not show a difference in the RA volume (*P* = 0.195). Additionally, the ablated area was significantly less in the termination group than in the non-termination group (1.97 ± 0.75 vs. 2.30 ± 0.73, *P* = 0.030). There was no significant difference in the ablated area in the termination group between patients who converted to SR and those who converted to AT/AFL (1.94 ± 0.83 vs. 2.00 ± 0.71, *P* = 0.814). The most common ablation site was the posterior wall, followed by the roof and the base of the left pulmonary vein. The most common termination site was the roof, the posterior wall, the anterior wall, and the base of the left pulmonary vein (detailed information was shown in Supplementary Figure 1). According to our protocol, all patients converted to AT/AFL received additional linear ablation after mMSE-guided ablation. Patients restored to SR after mMSE-guided ablation and those who did not achieve AF termination received additional termination linear ablation according to operators’ judgment. In non-termination group, 52 patients (74.29%) received linear ablation, similar to the percentage in termination group (30 patients, 78.95%, *P* = 0.588). Regarding the specific line, the percentage of mitral isthmus ablation, Cavo-tricuspid isthmus ablation, roofline ablation, and anterior line ablation did not differ between the non-termination group and termination group. Among patients with AF termination, patients converted to AT/AFL had a higher mitral isthmus and roofline ablation rate, while other lines’ ablation was similar in both subgroups. Lastly, we also collected procedure-related times. Total procedure time, fluoroscopy time, MSE ablation time, and total ablation time were significantly longer in the non-termination group than in the termination group, while mapping time and MSE calculation time showed no significant difference between the termination and the non-termination group. All above procedure-related time did not differ between subjects restored to SR and subjects converted to AT/AFL. However, termination time was significantly longer in patients restored to SR than in those converted to AT/AFL (*P* = 0.003).

**TABLE 2:** Acute procedure outcome and intraprocedural parameters of subjects.

Parameters	Non-termination group (*n* = 70)	Termination group (*n* = 38)				*P*-value*
			
		Total	Restored to SR	Converted to AT/AFL	*P*-value^†^	
			(*n* = 17)	(*n* = 21)		
LA mapping point number	609.93 ± 35.77	615.72 ± 32.73	620.46 ± 37.57	611.90 ± 28.61	0.430	0.409
RA mapping point number	356.80 ± 31.72	349.04 ± 26.02	354.06 ± 26.19	344.97 ± 25.78	0.291	0.199
LA volume (ml)	141.04 ± 31.54	123.13 ± 28.35	116.47 ± 26.60	128.52 ± 29.20	0.196	0.004
RA volume (ml)	69.84 ± 18.41	60.42 ± 15.93	64.18 ± 12.57	57.38 ± 17.92	0.195	0.009
Number of ablated areas	2.30 ± 0.73	1.97 ± 0.75	1.94 ± 0.83	2.00 ± 0.71	0.814	0.030
Linear ablation (%)	52 (74.29)	30 (78.95)	9 (52.94)	21 (100)	<0.001	0.588
MI ablation (%)	47 (67.14)	24 (63.18)	8 (47.06)	16 (76.19)	<0.001	0.677
CTI ablation (%)	24 (34.29)	11 (28.95)	4 (23.53)	7 (33.33)	0.508	0.571
Roof line ablation (%)	30 (42.86)	16 (42.11)	4 (23.53)	12 (57.14)	0.037	0.940
Anterior line ablation (%)	10 (14.29)	8 (21.05)	2 (11.76)	6 (28.57)	0.206	0.368
Total procedure time (min)	153.48 ± 12.03	147.05 ± 11.09	145.00 ± 11.21	148.70 ± 10.98	0.313	0.007
Fluoroscopy time (min)	11.4 ± 2.1	9.9 ± 2.6	9.9 ± 2.6	9.9 ± 2.7	0.977	0.002
Total ablation time (min)	62.13 ± 11.72	53.88 ± 8.32	52.32 ± 8.55	55.13 ± 53.76	0.307	<0.001
Termination time (min)^#^	–	33.07 ± 8.23	37.33 ± 8.75	29.62 ± 6.00	0.003	–
Total mapping time (min)	26.2 ± 4.0	26.1 ± 6.3	27.2 ± 5.2	25.2 ± 7.0	0.341	0.885
MSE calculation time (min)	14.7 ± 3.2	15.0 ± 4.0	14.7 ± 4.2	15.2 ± 4.0	0.712	0.607
MSE ablation time (min)	22.70 ± 7.92	12.58 ± 5.74	11.46 ± 5.69	13.48 ± 5.77	0.289	<0.001
Cycle length (baseline, ms)	153.41 ± 15.65	154.71 ± 14.15	153.58 ± 15.01	155.63 ± 13.73	0.663	0.672
Cycle length (after PVI, ms)	157.10 ± 14.35	159.00 ± 18.82	160.56 ± 18.90	157.74 ± 19.12	0.653	0.557
Cycle length (after MSE-guided ablation, ms)	170.84 ± 22.76	466.36 ± 265.52	753.23 ± 59.21	234.13 ± 32.26	<0.001	<0.001

*Comparison between non-termination group and termination group.

^†^Comparison between restored to SR group and converted to AFL/AT group.

^#^Termination time included the time from PVI to AF termination during mMSE-guided ablation, but not included the time for MSE mapping and calculation after PVI.

SR, sinus rhythm; AT, atrial tachycardia; AFL, atrial flutter; LA, left atrium; RA, right atrium; MI, mitral isthmus; CTI, Cavo-tricuspid isthmus.

### Multiscale entropy difference between ablation termination and non-termination group

**FIGURE 3:**
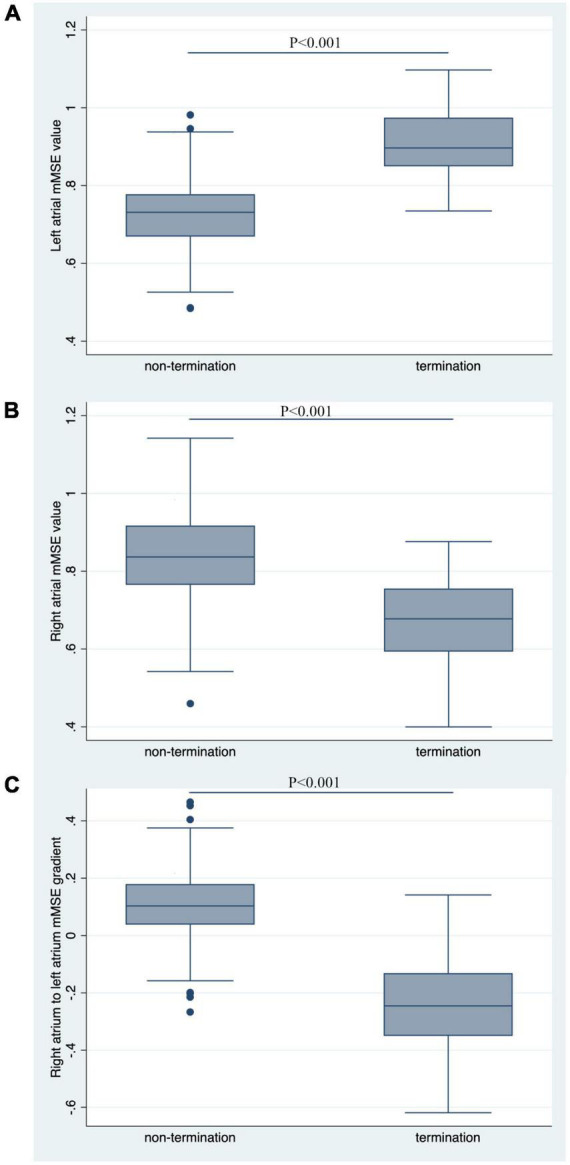
Comparison of mMSE values between groups. **(A)** The left atrial mMSE value was significantly higher in the termination group than in the non-termination group, with a *P*-value for comparison < 0.001. **(B)** Non-termination had a higher right atrial mMSE value than the termination group, with a *P*-value for comparison < 0.001. **(C)** RA-to-LA mMSE gradient was significantly higher in the non-termination group than in the termination group (*P* < 0.001).

The AF termination group had a significantly higher LA mMSE value than the non-termination group (0.89 ± 0.139 vs. 0.728 ± 0.125, *P* < 0.001, Figure 3A). In contrast, the mMSE value in RA was significantly higher in the non-termination group than in the termination group (0.833 ± 0.154 vs. 0.665 ± 0.187, *P* < 0.001, Figure 3B). As for the RA-to-LA mMSE gradient, the results demonstrated a positive gradient in the non-termination group (0.105 ± 0.180), a negative gradient in the termination group (−0.235 ± 0.256), and the difference between groups was significant (*P* < 0.001, Figure 3C).

### Follow-up outcome

**FIGURE 4:**
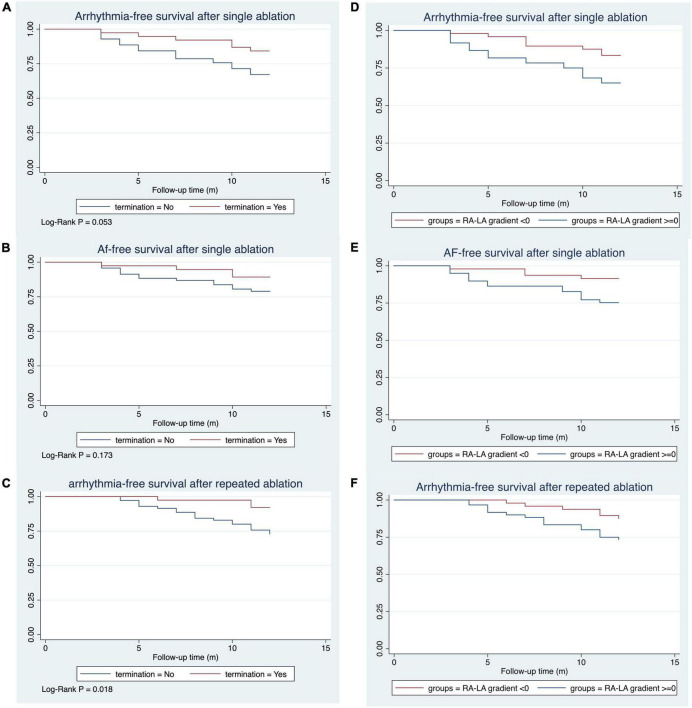
Kaplan–Meier curve for the survival rates after mMSE guided catheter ablation. The median follow-up time was 12.0 months. **(A)** The termination group had a substantially higher rate of arrhythmia survival after a single mMSE-guided catheter ablation, with a trend toward statistical significance (Log-Rank *P* = 0.053). **(B)** AF survival rate was higher in the termination group than in the non-termination group (Log-Rank *P* = 0.173). **(C)** Arrhythmia survival was significantly higher in the termination group than in the non-termination group (Log-Rank *P* = 0.018). **(D,E)** Subjects with a negative RA-to-LA mMSE gradient had a lower arrhythmia recurrence rate (Log-Rank *P* = 0.028) and AF recurrence rate (Log-Rank *P* = 0.032) than their counterparts after a single ablation process. **(F)** The negative RA-to-LA mMSE gradient group had higher rates of arrhythmia-free survival than the positive RA-to-LA mMSE gradient group after repeated ablation.

**FIGURE 5:**
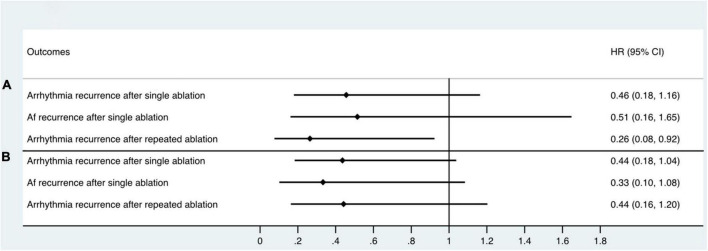
Cox proportional hazards regression model assessing the association between RA < LA mMSE gradient, intraprocedural termination, and follow-up outcomes. All models were adjusted for age, gender, AF duration, and LA volume. **(A)** RA < LA mMSE gradient reduced the risk of arrhythmia recurrence after single ablation by 54.4% (*P* = 0.101). Similarly, the risk of AF recurrence after single ablation was decreased to 51.5% in RA < LA mMSE gradient group than that in RA > LA mMSE gradient group (*P* = 0.264). Finally, RA < LA mMSE gradient group had a significant 73.6% risk reduction for arrhythmia recurrence after repeated ablation than RA > LA mMSE gradient group (*P* = 0.037). **(B)** Intraprocedural termination also showed associations with reduced risk of arrhythmia recurrence after single ablation, AF recurrence after single ablation, and arrhythmia recurrence after repeated ablation. However, the associations between intraprocedural termination and follow-up outcomes did not achieve statistical significance.

With a median follow-up time of 12.0 months, 29 patients (26.9%) had arrhythmia recurrence after single ablation (Figure 4). Among these patients, 18 patients had AF (62.1%), and 11 patients (37.9%) had AFL or AT recurrence. 6 of the 29 recurred patients were patients who converted to SR or stable AT/AFL during the ablation, while 23 were patients in the non-termination group (Log-rank *P* = 0.053, Figure 4A). Among the 18 patients with recurrent AF, 14 were in the AF non-termination group, and four were in the AF termination group (Log-rank *P* = 0.173, Figure 4B). Similarly, 2 of the 11 patients with AFL/AT recurrence were from the termination group, and the rest were from the non-termination group. When taking repeated ablation into account, only 3 out of 38 patients (7.89%) in the termination group had arrhythmia recurrence during the follow-up. In comparison, 19 out of 70 patients (27.14%) of the non-termination group had arrhythmia recurrence, and the difference between the groups was significant (Log-rank *P* = 0.018, Figure 4C). Our study observed similar KM results when dividing subjects into groups according to the RA-to-LA mMSE gradient. The group of negative RA-to-LA gradient showed a significantly lower rate of arrhythmia recurrence (16.67 vs. 35%, Log-Rank *P* = 0.028) and AF recurrence (8.33 vs. 23.33%, Log-Rank *P* = 0.032) after single ablation process. Regarding the arrhythmia-free rate after repeated ablation, the group of negative RA-to-LA mMSE gradient also displayed a higher rate than the group of positive RA-to-LA mMSE gradient (26.67 vs. 12.5%), but the difference only demonstrated a trend toward statistical significance (Log-Rank *P* = 0.062, Figure 4F). To further evaluate the independent association between RA-to-LA mMSE gradient, intraprocedural termination, and follow-up outcomes, our analysis employed Cox regression (Figure 5). All models were adjusted for age, gender, AF duration, and LA volume. The results were consistent with the KM curve. In Figure 5A, the results showed that a RA < LA mMSE gradient could reduce the risk of arrhythmia recurrence after single ablation by 54.4% (*P* = 0.101). Similarly, the risk of AF recurrence after single ablation was reduced to 51.5% in the RA < LA mMSE group than that in the RA > LA mMSE group, and the association was also insignificant (*P* = 0.264). Finally, the RA < LA mMSE group had a significant 73.6% risk reduction for arrhythmia recurrence after repeated ablation than the RA > LA mMSE group (*P* = 0.037). In Figure 5B, intraprocedural termination was associated with reduced risk of all three outcomes. However, none of the associations reached statistical significance.

### Complications

Procedure-related vascular complications occurred in 13 patients, with femoral hematoma in ten, A-V fistula in two, and pseudoaneurysm in one. Serious adverse events include one patient with pericardial effusion and one who suffered from a TIA. They were managed conservatively with no long-term sequelae. There was no occurrence of significant PV stenosis, other embolic complications, atrial-esophageal fistula, or death.

## Discussion

To the best of our knowledge, this is the first clinical study evaluating the feasibility, efficacy, and safety of MSE-guided driver ablation in patients with PsAF. Our main findings are as follows: (1) Ablation of AF driver guided by the novel MSE analysis mapping approach was associated with the successful ablation in a PsAF cohort; a negative baseline RA-to-LA mMSE gradient could associate with a higher rate of successful AF termination during catheter ablation; (2) Patients with a baseline negative RA-to-LA mMSE gradient could correlate with a higher rate of free from arrhythmias recurrence with repeated ablation; (3) Procedural AF termination could predict the long-term success rate with repeated ablation. Our findings still need larger studies with detailed information collection to validate.

### Driver mapping and ablation for persistent atrial fibrillation

The optimal ablation strategy beyond PVI for PsAF remains unclear (20). Additional anatomical-based ablation strategies have failed to show incremental advantage due to paying scant attention to the underlying mechanisms of complex wave propagation dynamics during AF. Recent developments in mapping tools and computational methods for advanced signal processing made novel strategies to identify atrial regions associated with AF maintenance (8, 21–23). A few prior studies have examined other techniques for AF driver identification to aid ablation (7, 8, 24–26). The CONFIRM trial studied ablation guided by focal impulse and rotor modulation, using a 64-pole basket catheter in the atrium to collect electrogram data for analysis and reported promising results (27). However, other groups have not replicated these results, most notably the larger REAFFIRM trial (28). The 252-electrode surface electrocardiography mapping vest is another technology used to map AF drivers. This non-invasive technology uses sophisticated computer back-extrapolation to deduce electric activity on the heart surface from signals collected from a multielectrode array on the chest (29). These approaches rely on calculating phase singularities to identify potential AF drivers rather than traditional mapping and electrogram processing techniques. They are also impeded by the lower spatial resolution of these technologies, which can cause drivers to be missed, incorrectly located, or artifactually created where they do not exist (12). Seitz et al. recently reported that regional spatiotemporal electrogram dispersion was associated with the presence of AF driver, and ablation of dispersion regions achieved a significantly higher AF termination rate and favorable outcome compared with the PVI ablation strategy (11). However, this method is too subjective in its judgment of potential and lacks quantitative standards.

In contrast, the novel approach guided by MSE Analysis uses high-density contact mapping and standard electrogram processing techniques to circumvent these limitations. The MSE approach was proposed using a coarse-grained time scaling procedure for a more robust determination of the complexity of time series data (30). This approach was introduced with time-averaged time series over multiple time scales for short time series analysis. Ravikumar et al. demonstrated that the MSE approach accurately identified the pivot point of both stationary and meandering rotors from both unipolar and bipolar iEGMs and performed marginally better for the multielectrode multi-spline simulations, that is, low spatial resolution (15). In this study, we reported the first-in-human results of the MSE approach to identify drivers for ablation in PsAF.

### Prediction of acute procedure outcome by complexity characteristics

Correlations between acute or long-term ablation outcomes and electrophysiological parameters have been studied, with longer AF cycle lengths (AFCLs), higher activation pattern recurrence, and lower AF complexity predicting favorable outcomes (31). We hypothesized that greater RA vs. LA MSE, or a positive RA-to-LA mMSE gradient, correlates with failure of left-sided ablation. The results confirmed our hypothesis. In our cohort, the termination group had a significantly higher LA mMSE value, a lower RA mMSE value, and a lower RA-to-LA mMSE gradient than the non-termination group.

The correlation between lower AF complexity and acute ablation success is consistent with previous studies reporting comparable results on complexity parameters based on DF, AFCL variability, and SampEn (31–33). The MSE approach correlates not only with AFCL variability but also with activation pattern variability, electrogram morphology variability, and fractionation, all of which have been associated with AF substrate (16). The association between the RA-to-LA MSE gradient at baseline and AF recurrence on follow-up indicates differences in the underlying AF-maintaining substrate. Unlike AFCL, DF, and SampEn, MSE is likely sensitive to different electrogram characteristics, and therefore better at predicting acute and long-term ablation rhythm outcomes. This study suggested a readily measurable intra-procedural predictor (LA > RA MSE gradient) of left-sided ablation success after PVI.

The findings from our work were consistent with the conclusion of a published study. Johner et al. (31) have identified that higher RA electrical complexity than CS electrical complexity could predict unfavorable acute outcomes of left-side PsAF ablation (31). In their results, PsAF patients with successful ablation had a significantly lower positive RA-to-CS sample entropy (SampEn) gradient than those with failed ablation (19 vs. 48%, *P* = 0.015). Similarly, our results suggested that PsAF patients with AF termination had a significantly higher rate of negative RA-to-LA MSE gradient than those without AF termination. However, our study still had differences with Johner et al.’s study. First, our study used MSE instead of SampEn to assess the degree of electrical complexity. MSE is a modified scale of entropy compared to SampEn; it assesses the complexity more comprehensively *via* multiple scales; MSE improved the accuracy of the estimation of complexity by implementing a moving average time series estimate (34). Second, Johner et al.’s study only used a fixed CS electrode and a fixed 10-electrode catheter in RA to reflect the electrical signal complexity in atria. Our study used signals collected by the PentaRay catheter in the atria instead. Therefore, we believe our data reflected the complexity of the atria more accurately. Most importantly, our study used the mMSE value to guide the location of ablation in addition to PVI. In other words, MSE was not only a predictor of the outcomes of the left-side ablation, but also a potential tool for achieving a novel ablation strategy. But in Johner et al.’s study, they merely used SampEn to predict the outcomes of conventional ablation strategies.

It is necessary to discuss the rationale for the association between the RA-to-LA mMSE gradient and the AF termination rate. The whole study and the mMSE-based ablation were based on the theory that the AF was maintained by the rotor (3, 4). According to the re-entry nature of the rotor, the closer to the core, or the phase singularity of the rotor, the more chaotic and high-frequency electric signals were recorded. On the contrary, the electrode will detect relatively low-frequency and regular electric signals. The MSE technique was designed to measure the degree of signal complexity. Therefore, if the core of rotors exists in the LA, the top five mMSE values in LA will indicate the location of the core in the LA, and the top five mMSE values in RA could only indicate the sites with moderate signal complexity in the relatively outer layer of the rotor. In this scenario, the RA-to-LA mMSE gradient was negative, and if we ablate the sites with the highest mMSE values in LA, the AF termination will probably occur. However, suppose the core of rotors exists in the RA. In that case, the top five mMSE values in LA will indicate the sites with moderate signal complexity in the relatively outer layer of the rotor. Still, the top five mMSE values in RA could indicate the location of the core in the RA. Under this condition, RA-to-LA mMSE gradient was positive, and if we ablate the sites with the highest mMSE values in LA, the possibility of AF termination is low.

In Table 2, the non-termination group had a significantly large volume of LA than the termination group, suggesting the association between increasing LA size and increasing AF drivers. The observed association could be explained by the primary two points: First, a larger LA size provides a large area for multiple rotors to maintain; Second, a larger LA size is always accompanied by significant atrial fibrosis, which prolongs potential transduction, reduces the areas needed to maintain drivers, and influences the location of the phase singularities, and thereby increasing the number of AF drivers (35, 36).

### Multiscale entropy gradient, acute atrial fibrillation termination, and relation with long-term outcome

For driver-based AF ablations, termination of AF during ablation has been proposed as an indicator of successful AF substrate modification and therefore an ablation endpoint, but evidence supporting this use is still inconclusive. While AF termination during ablation appeared to be a strong predictor of success in several studies (19, 37), the rate of PsAF termination by ablation varied significantly between different approaches and centers, and studies such as the IU-FIRM study failed to demonstrate an association between the termination of AF during FIRM ablation and long-term freedom from recurrent AF (38). Inconsistent with their findings, we found that patients with acute AF termination could have better outcomes than those without AF termination. During the follow-up, the termination group had a higher rate of freedom from arrhythmia recurrence after a single ablation process than the non-termination group, and the difference showed a trend toward statistical significance. Similarly, the AF recurrence rate was also lower in the termination group, but the difference with the rate in the non-termination group displayed no trend toward significance. Consistently, after dividing subjects according to their RA-to-LA mMSE gradient, we observed that subjects with negative RA-to-LA mMSE gradient had significantly lower rates of arrhythmia and AF recurrence, implicating the potential impact of RA-to-LA mMSE gradient in predicting arrhythmia recurrence after catheter ablation. The Cox regression demonstrated similar results to the survival curve. After adjusting age, gender, AF duration, and LA volume, our results identified that the RA < LA mMSE gradient was associated with arrhythmia and AF recurrence rate after single ablation, but the associations were insignificant. However, because of the small sample size and the low incidence of recurrence, we believe the insignificance mainly resulted from a lack of statistical power. Finally, the arrhythmia recurrence rate after repeated ablation was significantly lower in the RA < LA mMSE gradient group than the RA > LA mMSE gradient group, suggesting the positive prognostic value of the mMSE-guided successful ablation. We propose that the discrepancy in findings might be caused by the following factors: (1) The heterogeneity of the study subjects is likely to affect the results. They evaluated a mixed cohort of paroxysmal and persistent AF. For paroxysmal AF, in patients whose AF is initiated by pacing maneuvers, we cannot exclude the possibility that ablation may lead to a serendipitous termination. (2) Different mapping methods in driver detection contributed to different results. Either a false negative or false positive may cause the leading AF driver to be missed.

Our long-term follow-up results also showed consistency with Johner et al.’s study (31). In their study, successful ablation and a negative RA-to-CS SampEn gradient were associated with fewer AF recurrences and freedom from AF. Our data also supported the significant association between termination, a negative RA-to-LA mMSE value, and a higher rate of freedom from arrhythmia recurrence.

It is necessary to discuss the applicating condition of the MSE-guided ablation. In Figure 3, we observed a negative RA-to-LA gradient of mMSE gradient in the termination group, and a positive gradient of mMSE gradient in the non-termination group. If we put LA and RA mMSE values together and take the top 5 from the pool, the target area would distribute in one atrium, possibly LA for the termination group and RA for the non-termination group. If all the top 5 mMSE values are located in RA, we believe we still cannot leave LA alone and ablate in RA only. Because currently the MSE technology is still in the developing stage. Even with promising results in the current study, the ablation strategy should not deviate from the common path, LA ablation is still fundamental for the PsAF ablation, and the MSE-guided ablation could only provide a method to facilitate and improve the ablation strategy selection.

### Complications

Regarding safety endpoints, our study observed 2 cases of serious complications, one with pericardial effusion, the cause of this case was inappropriate atrial septum perforation and was not correlated with the ablation process. One with a TIA. However, this patient had a TIA history before the ablation and was reported to have severe carotid artery calcification. Therefore, the possibility that the ablation process directly led to TIA, in this case, is relatively low. Our study showed a relatively higher rate of vascular complications. The reason is that we routinely used intracardiac echocardiography to replace transesophageal echocardiography to exclude intracardiac thrombi, and the coronary sinus electrode was planted through femoral vein access. Therefore, a total of four femoral vein puncture was conducted for every patient. In the early stage of switching to this strategy, the incidence of vascular complications was relatively high, and this study was conducted during this stage, after months of practice, the rate of vascular complications returned to a lower range.

## Limitations

The study has significant limitations. Firstly, the study used a single-arm design, making the comparison with other ablation strategies difficult, including the PVI-only plus electrical cardioversion strategy. Therefore, from our current work, we cannot answer whether targeting MSE will reduce AF recurrence with less additional AFL/AT compared to other established methods. Furthermore, whether MSE analysis and Driver mapping will significantly increase the overall procedure time compared with PVI only or PVI plus other strategies can also not be concluded based on our study. However, because our study is the first study employing MSE analysis to guide the ablation strategy for PsAF, we believe that exploring the feasibility, efficiency, and safety of the MSE-guided ablation through a limited sample size is more important than comparing the MSE-guided ablation with other ablation strategies through a randomized controlled trial (RCT) with a larger sample size in the current work. By elucidating the feasibility, efficiency, and safety of the MSE-guided ablation first, we can avoid potential ethic problems, and provide data for to design RCT to compare the MSE-guided ablation with other catheter ablation strategy. Nevertheless, due to the observational design of our current study, more studies with double or three-arm randomized designs should be conducted to address this question. Secondly, although the MSE technique was validated using optical mapping in animal models and computer-simulated mapping, mapping catheters in current use has a lower resolution which may affect the accuracy of calculated MSE. Furthermore, because the current workflow of MSE calculation involved a series of manual work, including export, import, and annotation in the CARTO system, the whole process was time-consuming. And the current MSE analysis cannot be implemented into the CARTO system, which caused a time delay between mapping and ablation. Therefore, we used the mMSE value of each mapping site rather than MSE value of each electrode pair to construct the 3D MSE map. This action would compromise the resolution of the 3D MSE map and subsequently jeopardize the efficacy of MSE-guided ablation, but it can minimize the MSE calculation process to an acceptable range. More integrated workflow and software are needed to accelerate the MSE calculation process and improve the efficacy of MSE-guided ablation in the future. Thirdly, due to the limited area of recording by the Pentaray catheter, temporal and spatial variation of the rotors could be underestimated under the current hardware condition, thereby limiting the efficiency of the mMSE mapping system. Therefore, development of suitable hardware is needed to improve the performance of the mMSE mapping system. Fourthly, as a pilot study, our study was designed to evaluate the efficacy and safety of MSE-guided catheter ablation in human subjects. We only enrolled 108 patients with PsAF, and follow-up results revealed a lack of statistical power. Due to this small sample size, we were unable to compare MSE and other traditional methods like DF. Additionally, we cannot calculate DF in the current workflow due to software and hardware issues. Therefore, more studies with larger sample sizes, more powerful software and hardware supports, and more detailed information collection are needed to confirm our findings. Fifthly, as with other observational research, unrecorded variates can also cause residual confounding, thereby introducing bias into our results. For example, we could not adjust the confounding effect caused by the remodeled substrate because we did not quantify the size of the remodeled substrate. This may compromise the value of the finding that mMSE-guided ablation could provide useful information about the ablation sites. However, even in patients with a low degree of substrate remodeling, AF termination is not likely to be achieved by ablating 1.97 ± 0.75 atrial areas without any electrogram-based guidance. The common strategy is based on linear ablation if there is no electrogram-based guidance. Therefore, even with the confounding effect caused by the remodeled substrate, our results still suggested but did not conclude that the mMSE-guided ablation could provide some useful guidance for the ablation sites. However, this finding should be verified in future studies which record detailed information about the size of the remodeled substrate. Sixthly, our current ablation strategy did not include any RA ablation. A positive RA-to-LA mMSE gradient was suggestive of RA-originated AF from our results. RA ablation in these subjects may improve the total success rate of procedural AF termination. Future studies with ablation strategies including RA ablation are needed to address this point. Sixthly, the current study focused on the performance of mMSE-guided ablation in PsAF patients with mild-to-moderate LA lesions, we excluded patients with LAD equal to or more than 60 mm. Hence, our results could not provide evidence for the usefulness of mMSE-guided ablation in PsAF patients with severe LA lesions. More studies are needed to explore this point. Lastly, this study also did not include paroxysmal AF patients. Therefore, the current results cannot be extrapolated to all the AF population. Despite these apparent limitations, the initial outcomes are favorable in this refractory population of patients with PsAF.

In conclusion, our results suggest that the MSE analysis-guided driver ablation in addition to PVI for PsAF could be feasible, efficient, and safe. A negative RA-to-LA mMSE gradient before ablation was predictive of successful AF termination and freedom from AF. The RA-to-LA MSE gradient may be useful for guiding ablation strategy selection.

## Data availability statement

The raw data supporting the conclusions of this article will be made available by the authors, without undue reservation.

## Ethics statement

The studies involving human participants were reviewed and approved by the Institutional Review Board/Ethics Committee of Shanghai Chest Hospital (KSY21312). The patients/participants provided their written informed consent to participate in this study.

## Author contributions

W-RS and S-HW collected the study data, performed the statistical analysis, and wrote the manuscript. G-CZ designed the workflow of MSE-guided driver ablation. KX, W-FJ, and YZ performed the catheter ablation and wrote part of the method section. MQ and XL designed the study protocol and reviewed the manuscript. All authors contributed to the article and approved the submitted version.

## Abbreviations

AAD: antiarrhythmic drug
AF: atrial fibrillation
AFCL: atrial fibrillation circle length
AFL: atrial flutter
AT: atrial tachycardia
CTI: Cavo-tricuspid isthmus
CS: coronary sinus
DF: dominant frequency
iEGMs: intracardiac electrograms
KM: Kaplan–Meier
LA: left atrium
LVEF: left ventricular ejection fraction
MI: mitral isthmus
MSE: multiscale entropy
mMSE: mean multiscale entropy
PsAF: persistent atrial fibrillation
PV: pulmonary vein
PVI: pulmonary vein isolation
RA: right atrium
SR: sinus rhythm
TIA: transient ischemic attack.
