# Supplementary material for: A novel approach for quantitative electrogram analysis for driver identification: Implications for ablation in persistent atrial fibrillation
Source: Front Cardiovasc Med. 2022 Dec 1;9:1049854. doi: 10.3389/fcvm.2022.1049854 (PMC9751199; doi:10.3389/fcvm.2022.1049854)
Supplement: Supplementary file 1 [file Data_Sheet_1.pdf]

Supplementary Materials

Supplementary Figure 1

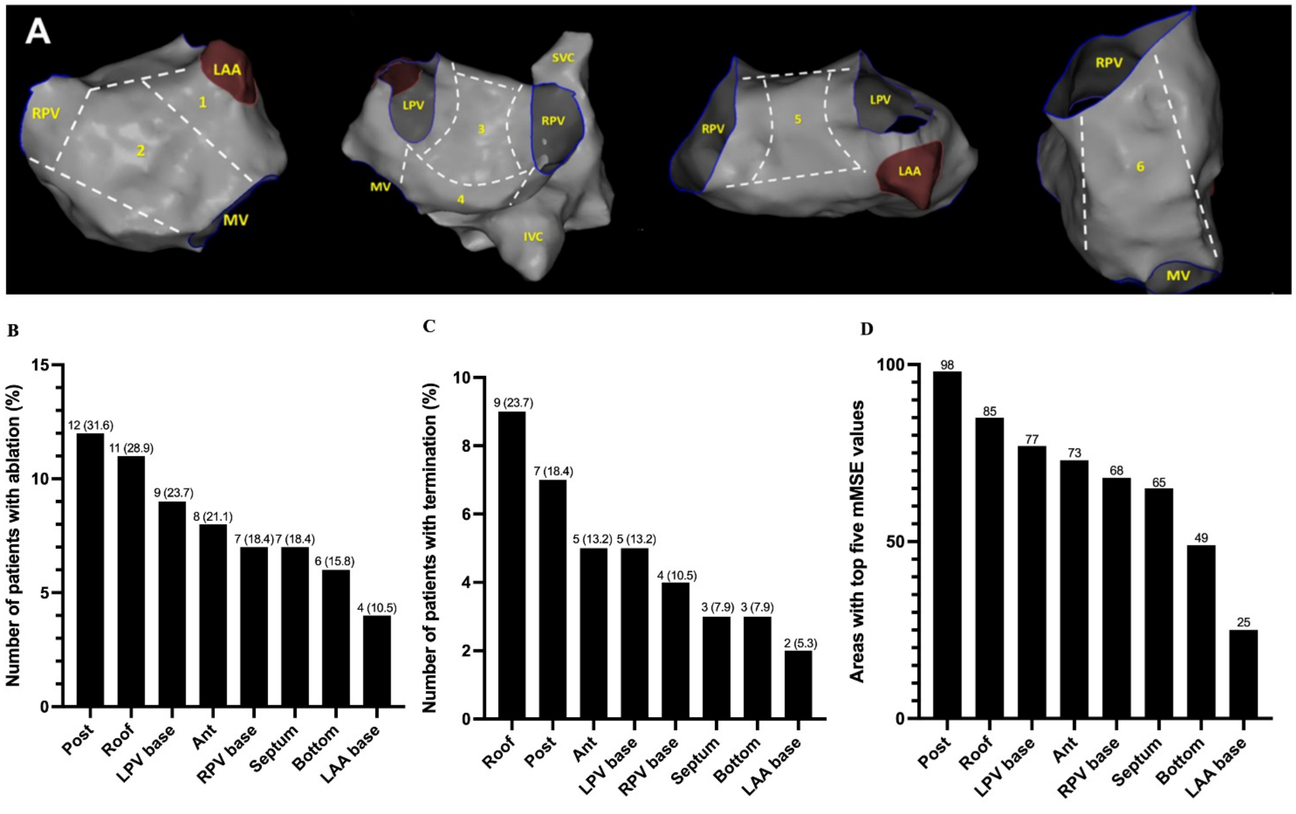

Figure S1. Distribution of ablation sites and termination sites in left atrium.
(A). Left atrium was distributed into 8 areas manually. 1. LAA basal; 2. Anterior wall; 3. Posterior wall; 4. Bottom; 5. Roof; 6. Septum; 7. Base of right pulmonary vein; 8. Base of left pulmonary vein. (B). Distribution of ablation sites in the left atrium. (C). Distribution of termination sites in the left atrium. (D). Distribution of the top five mMSE areas in the left atrium.
Abbreviations: Post: posterior wall; Ant: anterior wall; LPV: left pulmonary vein; RPV: right pulmonary vein; LAA: Left atrial appendage.

Supplementary Methods

Detailed description of the MSE calculation workflow

1. Mapping Data Collection

Study participants were recruited from the consecutive persistent AF patients in Shanghai Chest Hospital from November 2019 to December 2020. Every patient enrolled in the current study provided an informed consent before any operation.

The cardiac electrophysiologists accomplished the data collection process in the cardiac catheterization laboratory in the department of cardiovascular medicine, Shanghai Chest Hospital. Three expert cardiac electrophysiologists, namely Dr. Xu Liu, Dr. Mu Qin, and Dr. Shaohui Wu, participated in this study. All of them are experienced in the field of ablation for AF and other arrhythmias. The CARTO (Biosense Webster, Inc., Diamond Bar, California) system, with a sensor position accuracy of 0.8mm and 5°, was utilized to perform the electro-anatomical mapping and the subsequent ablation. The PentaRay NAV catheter (Biosense Webster, Inc., Diamond Bar, California) was used as the mapping catheter for every patient in our study. The system recorded both unipolar and bipolar iEGMs at a frequency of 1000Hz, allowing the electrophysiological information to be coded with color and attached to the reconstructed model of LA and RA. Regarding the reconstruction of the real-time 3D geometry of LA and RA, the three expert cardiac electrophysiologists performed the mapping procedure for persistent AF patients under a standard procedure protocol for AF mapping. The septum was mapped from both LA and RA. An automated point collection mode (ConfiDENSE Continuous mapping) was used to avoid inappropriate point collection when electrodes were not at the inner face of the atria. This mode allows point collection only when a contact force equal to or more than 2-g was detected at the tip of the catheter. 5-10 s electrograms were collected at each point along with the surface ECG. As a result, they constructed a CARTO database consisting of iEGMs from both atria of each patient who underwent electro-anatomical mapping for ablation. Multiple points were taken to create geometry with a fill threshold of 20 with uniform distribution across both chambers.

2. Data extraction and analysis

When the mapping was completed and the iEGMs data stored in the CARTO database, iEGMs data from both atria were extracted from the CARTO database manually and exported in SAV format. For MSE analysis, bipolar electrograms recorded by ten specific electrode pairs in the PentaRay catheter were needed for every mapping site. The ten electrodes pairs were electrodes pair 1-2, 3-4, 5-6, 7-8, 9-10, 11-12, 13-14, 15-16, 17-18, 19-20. Due to the restriction of the CARTO system, a maximum of 2.5 seconds of electrograms at each site were exported along with the surface ECG. The raw iEGMs were then imported into a customized analyzing platform (Eclipse, solvusoft, Las Vegas, USA.). After that, the desired ten bipolar electrogram signals from the PentaRay catheter and surface ECG signals were captured with the assistance of artificial intelligence. After a pre-processing noise canceling, the MSE values were calculated based on the ten bipolar electrograms and sorted automatically. Finally, the results were exported in the format of TXT.

2.1 Pre-processing noise canceling

Although the bipolar signals used for data analysis are less affected by the signal noise, a total absence of baseline noise and ventricular far-field noise seems impossible since the noise depends on the electrode spacing and the timing of the ventricular activity during the iEGMs collecting process. Therefore, preprocessing the bipolar iEGMs is essential before any signal processing begins.

There are three common noises contamination types:

- Baseline wander noise which leads to the drifting isoelectric line
- High frequency noise including the 60Hz power line interference noise
- Ventricular far-field noise caused by the close distance to the ventricles.

1) Baseline wander noise canceling

   Baseline wander noise is characterized by the low frequency movement of the isoelectric line in the electrocardiography. It can be caused by the patient's movement, such as chest movement during breathing or coughing, interference, poor electrode contacts, or drift of the reference potential. The frequency of baseline wander noise is always below 2 Hz. Discrete Wavelet Transform (DWT), by using Daubechies wavelet of order 11 and wavelet filter banks, can be used to cancel the baseline wander noise (1).

2) High frequency noise canceling

   Multiple sources, such as power line interference, other electronic noise sources from the mapping system, electromyogram noise, and high frequency motion artifact noise, can contaminate the iEGMs with high frequency noise. Canceling these high frequency noises is the basis for accurate recognition of the atrial electrical signals. The IIR Butterworth low pass filter with a cutoff between 30-400 Hz can be employed to cancel high frequency noise.

3) Ventricular Far Field (VFF) noise canceling

   When the iEGMs are collected proximal to the ventricles, they can be contaminated by the ventricular signals, causing VFF. This phenomenon is usually observed in specified areas such as the inferior anterior atrial wall, the inferior posterior atrial wall, or the coronary sinus (1). Accordingly, canceling the VFF noise is an inevitable step before further signal processing.

   The VFF noise canceling can be accomplished through Principal Component Analysis (PCA). During the process, body surface ECG is required to obtain accurate QRS time stamps according to the ventricular activity (2-4). Lead II ECG will be a favorable choice for obtaining clean ECG and obtaining accurate QRS proximity. The first step to cancel VFF is to segment the contaminated iEGMs based on the QRS timestamps from Lead II ECG to isolate the VFF from the iEGMs in a time window of 180ms around the estimated peak of the R-wave. Then the PCA components can be estimated. Based on the ventricular to atrial activity signal ratio (VASR), the criterion for deleting the required number of PCA components to remove VFF can be established (1). Finally, an inverse PCA operation is conducted to reconstruct iEGMs without VFF.

In total, the raw iEGMs were filtered between 30-400Hz. Therefore, the data used in the analysis were free of noise and signal loss. To limit the signal frequencies to the physiological ranges, an additional filter of 330Hz was applied to the remaining iEGMs. After canceling the three major types of signal noise, the preprocessed iEGMs can be used for MSE analysis to locate the pivot point of the rotor.

2.2 Multiscale entropy calculation

The challenge with the analysis of short time series data is that the complexity of the data may not embed in the raw signal. Therefore, most approaches only show excellent work when dealing with long time series data. However, the collection of the iEGMs determines that it is a short time series data and cannot be processed by most conventional approaches. The MSE approach was proposed to use coarse-grained time scaling procedure for more robust calculation of the complexity

of time series data (5). The coarse-graining procedure may result in invalid entropy values estimation for shorter time series; and this limitation was addressed by implementing a moving average time series estimate (6). However, the moving average was only conducted forwardly, which can lead to an underestimation of the complexity information that is present in the time series data. To address this problem, a nearest neighbor moving average kernel was employed to capture better the complexity of non-stationary and non-linear short time series data. The concept of "memory" refers to considering the past and future time series value while computing the nearest neighbor moving average for time series data. Therefore, the time scale factor "τ" represents time scaling in both forward and reverse directions concerning a particular time point. Once this new time series is derived, an MSE estimate can be obtained by calculating the entropy of the new time series sample over multiple time scales to fully capture the intrinsic complexity of non-stationary and non-linear time series data.

The algorithm for the calculation of MSE used in our study was not available to the public. Here we describe the theory of MSE calculation (7). The conceptional steps were listed below:

Let $x = \{X_1, X_2, X_3 \ldots X_N\}$ represent the electrogram time series of length N.

1) According to the following formula, a nearest neighbor moving-averaged time series z^τ is computed for the chosen time scale factor 'τ':

$$z_j^\tau = \frac{1}{(2\tau+1)} \sum_{i=j}^{2\tau+1} x_i \qquad \text{(Eq.1)}$$

where $1 \leq j \leq N-\tau$ and $i = 1,2,3, \ldots N$; Figure 1 shows the schematic to obtain the nearest neighbor moving window averaging approach to obtain the new time series.

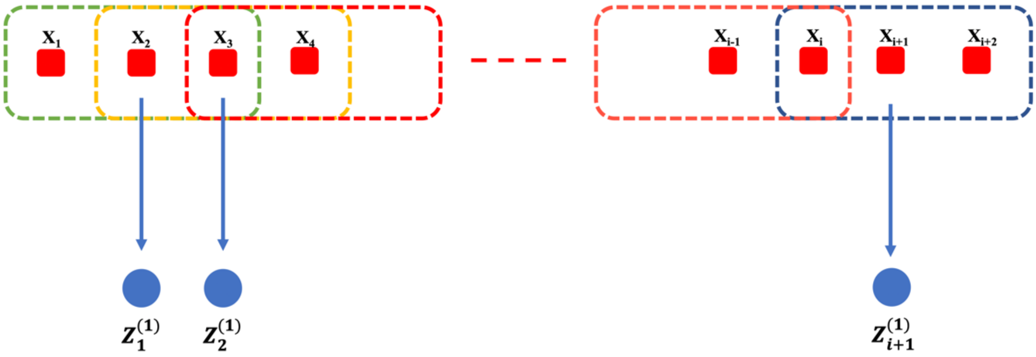

Figure 1: Schematic illustration to produce nearest neighbor moving average time series with scale factor τ=1 for the MSE algorithm. Blue squares represent raw time series data and red dots represent the nearest neighbor moving-averaged time series from which MSE is obtained. Brown square represent the moving window averaging kernel for the raw second time point (X_2), that Averages one neighbor on both sides with τ=1 to produce the first new time series point $Z_1^{(1)}$. Similarly green square produces $Z_2^{(1)}$ and so on (orange square) with the blue square producing the last times series point $Z_{i+1}^{(1)}$.

2) At each specific τ, template vectors $y_k^m(\delta)$ with dimension m and delay δ are constructed from z^τ (see Eq. 1) as the following:

$$y_k^m(\delta) = \{Z_k Z_{k+\delta} \ldots \ldots Z_{k+(m-1)\delta}\} \qquad \text{(Eq.2)}$$

Where $1 \leq k \leq N-m\delta$;

3) Using the infinity norm as listed below, the Euclidean distance d_{ij}^m for each pair of template

vectors $\{y_i^m, y_j^m\}$ is calculated:

$$d_{ij}^m(\delta) = \|y_i^m(\delta) - y_j^m(\delta)\|_\infty \qquad \text{(Eq.3)}$$

4) Based on a pre-defined tolerance threshold r, matched template vector pairs $\{y_i^m, y_j^m\}$ are computed as:

$$d_{ij}^m(\delta) \le r \qquad \text{(Eq.4)}$$

In this work, the value for 'r' is chosen to be 0.2 times the standard deviation of the raw time series χ. The delay factor δ is chosen to be 1. n (m, δ, r) denotes the total number of matched template vectors.

Steps 2-4 are then repeated for m+1 dimension, and the total number of matched template vectors being computed is denoted by n (m+1, δ, r).

Finally, the MSE is calculated by using the following formula:

$$MSE\ (x, m, \delta, r) = -\ln\frac{n(m+1,\delta,r)}{n(m,\delta,r)} \qquad \text{(Eq.5)}$$

According to the above conceptional steps, we customed a speciated software based on JAVA language to calculate the MSE value in each electrical pair of each spatial site.

3. Patient-specific 3D MSE map construction

In our current study, we used the mean MSE (mMSE) for each mapping site instead of the MSE value of each electrical pair in the mapping site to construct the 3D MSE map. mMSE was calculated as the mean of the MSE values of the 10 electrode pairs. By doing this, we will compromise the resolution of the final 3D MSE map. However, this action will shorten the time used in the whole process of 3D MSE map construction to an acceptable range.

The mapping sites were ranked descending and automatically by the software according to the mMSE value. The top five mMSE values at each atrium were inversely inputted into the CARTO system to create a color map attached to the anatomical model, which is called the patient-specific 3D MSE map. The highest mMSE value was highlighted as red, while the lowest mMSE value was labeled as purple. Therefore, electrophysiologists can locate the presumed rotor core according to the spatial location of the red areas.

In the current study, we did not use the data from the included 108 patients to validate the reproducibility of MSE analysis. However, prior to the beginning of this study, we used 6 PsAF patients to test the reproducibility of MSE analysis. In these patients, both atria were mapped twice with an interval equal to or more than 20 minutes. The results revealed that the MSE analysis showed an excellent reproducibility for stationary rotors (manually defined according to the procedure published by Seitz et al. (8)). On the contrary, the MSE reproducibility for meandering rotors was limited.

References:

1. Schilling. Analysis of Atrial Electrograms. *Karlsruhe transactions on biomedical engineering* (2012).

2. Arunachalam SP, Brown LF. Real-Time Estimation of the Ecg-Derived Respiration (Edr) Signal Using a New Algorithm for Baseline Wander Noise Removal. *Annu Int Conf IEEE Eng Med Biol Soc* (2009) 2009:5681-4. Epub 2009/12/08. doi: 10.1109/iembs.2009.5333113.

3. Brown LF, Arunachalam SP. Real-Time T-P Knot Algorithm for Baseline Wander Noise Removal from the Electrocardiogram - Biomed 2009. *Biomed Sci Instrum* (2009) 45:65-70. Epub 2009/04/17.

4. Brown LF, Arunachalam SP. Real-Time Estimation of the Ecg-Derived Respiration (Edr) Signal

- Biomed 2009. *Biomed Sci Instrum* (2009) 45:59-64. Epub 2009/04/17.
5. Costa M, Goldberger AL, Peng CK. Multiscale Entropy Analysis of Complex Physiologic Time Series. *Phys Rev Lett* (2002) 89(6):068102. Epub 20020719. doi: 10.1103/PhysRevLett.89.068102.
6. Borin AMS, Jr., Silva LEV, Murta LO, Jr. Modified Multiscale Fuzzy Entropy: A Robust Method for Short-Term Physiologic Signals. *Chaos* (2020) 30(8):083135. Epub 2020/09/03. doi: 10.1063/5.0010330.
7. Arunachalam SP, Kapa S, Mulpuru SK, Friedman PA, Tolkacheva EG. Improved Multiscale Entropy Technique with Nearest-Neighbor Moving-Average Kernel for Nonlinear and Nonstationary Short-Time Biomedical Signal Analysis. *J Healthc Eng* (2018) 2018:8632436. Epub 20180305. doi: 10.1155/2018/8632436.
8. Seitz J, Bars C, Théodore G, Beurtheret S, Lellouche N, Bremondy M, et al. Af Ablation Guided by Spatiotemporal Electrogram Dispersion without Pulmonary Vein Isolation: A Wholly Patient-Tailored Approach. *J Am Coll Cardiol* (2017) 69(3):303-21. Epub 2017/01/21. doi: 10.1016/j.jacc.2016.10.065.

Case presentation

Here we present a case who achieved successful AF termination through mMSE-guided ablation. The patient was a 62-year-old male. The main symptom was paroxysmal palpitation for about one year, aggregating after exertion. The patient was identified with AF by EKG and classified as PsAF through multiple Holter examination during the follow-up. The AF could not be terminated through receiving oral amiodarone, and the patient reported exacerbated symptom during the follow-up. Therefore, the patient was recruited into our study for radiofrequency ablation. Pre-procedural echocardiography showed the anteroposterior LA diameter was 46mm, other structural diameters remained in normal range. The pre-procedural EKG is displayed at below:

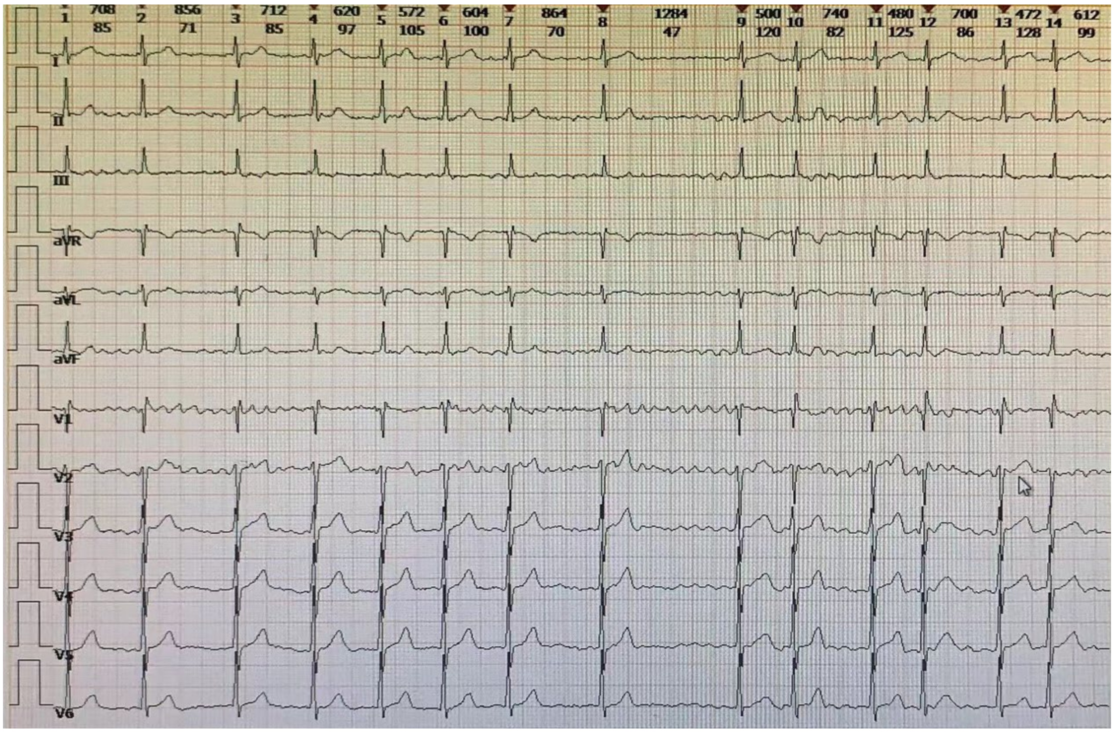

Then the patient received mMSE-guided AF ablation. The LA volume constructed in the CARTO 3 system was 131 cm^3. The condition before PVI was displayed as below:

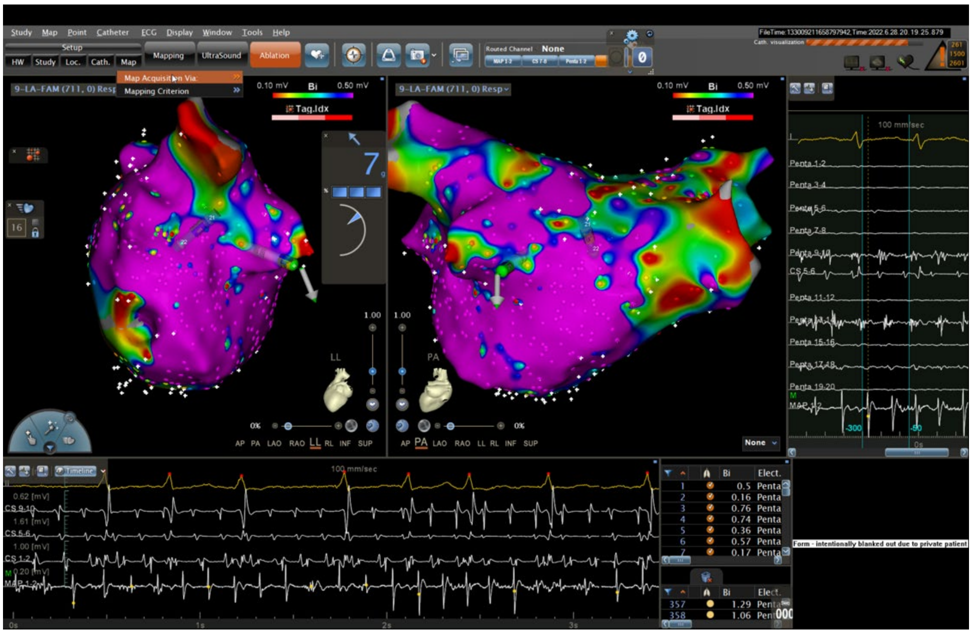

PVI was conducted as the first step of ablation.

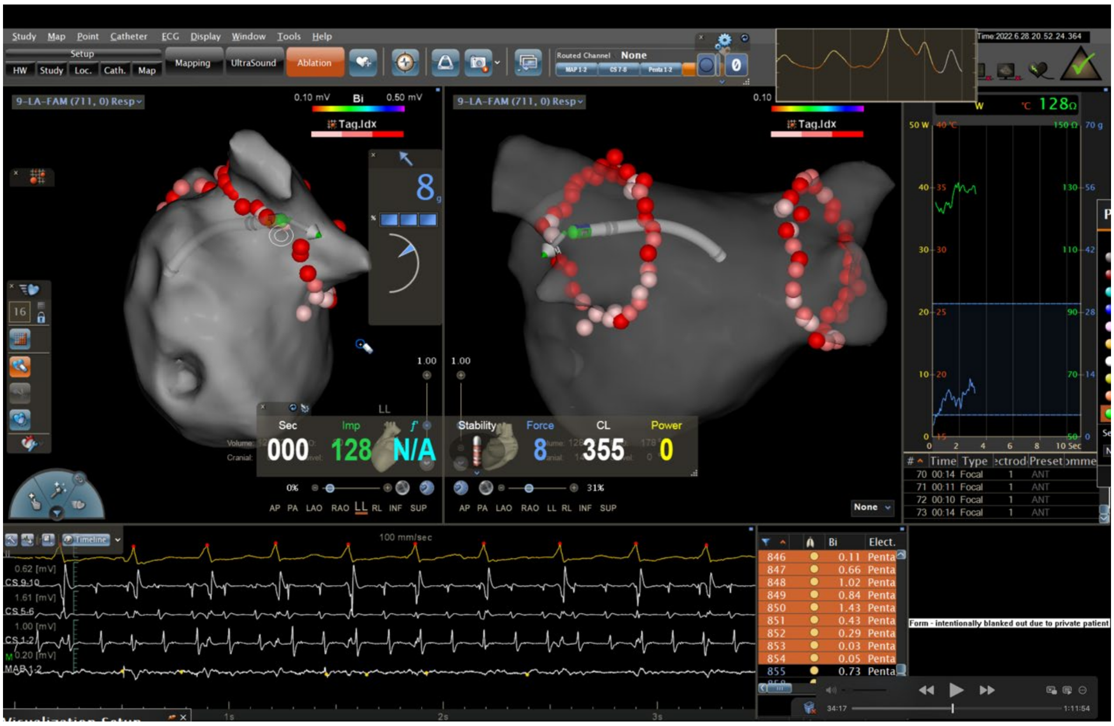

Then the RA was mapped, and the LA was remapped. Subsequently, CARTO 3 data were extracted, MSE values were calculated, and the 3D LA mMSE map was constructed. The MSE calculation process was conducted through a customed software as displayed below (use data from LA as example):

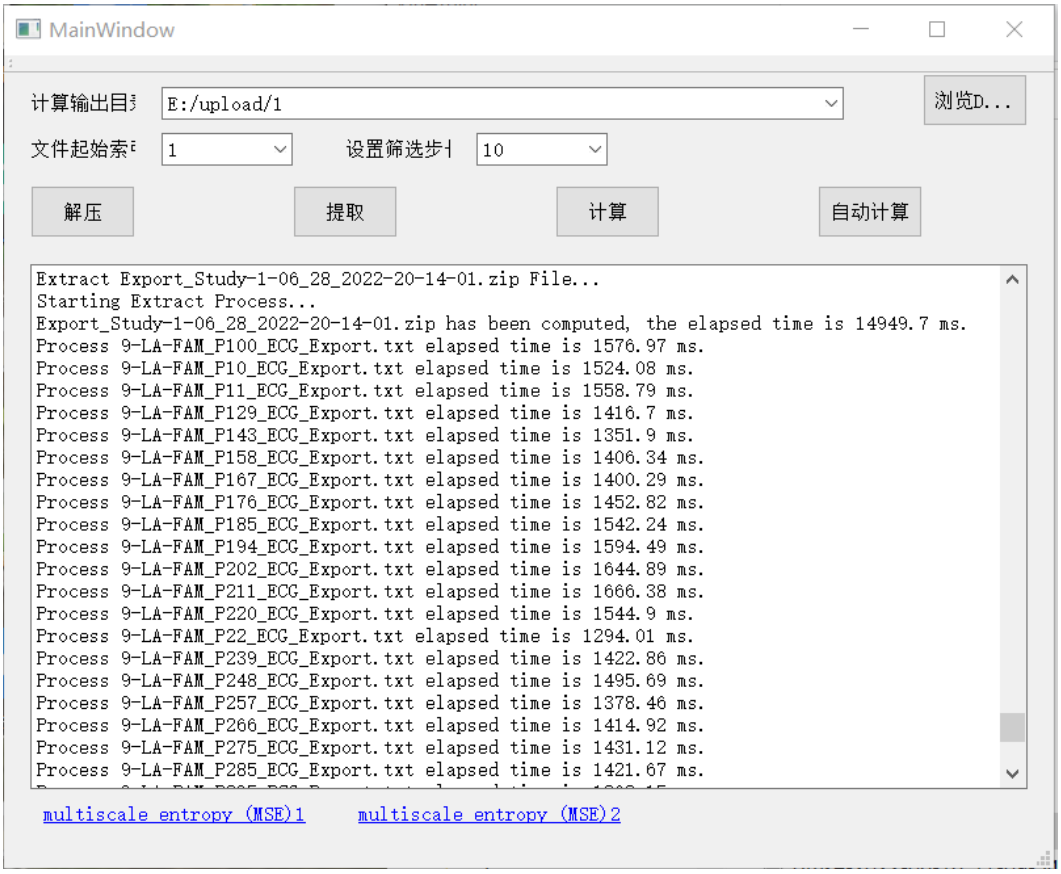

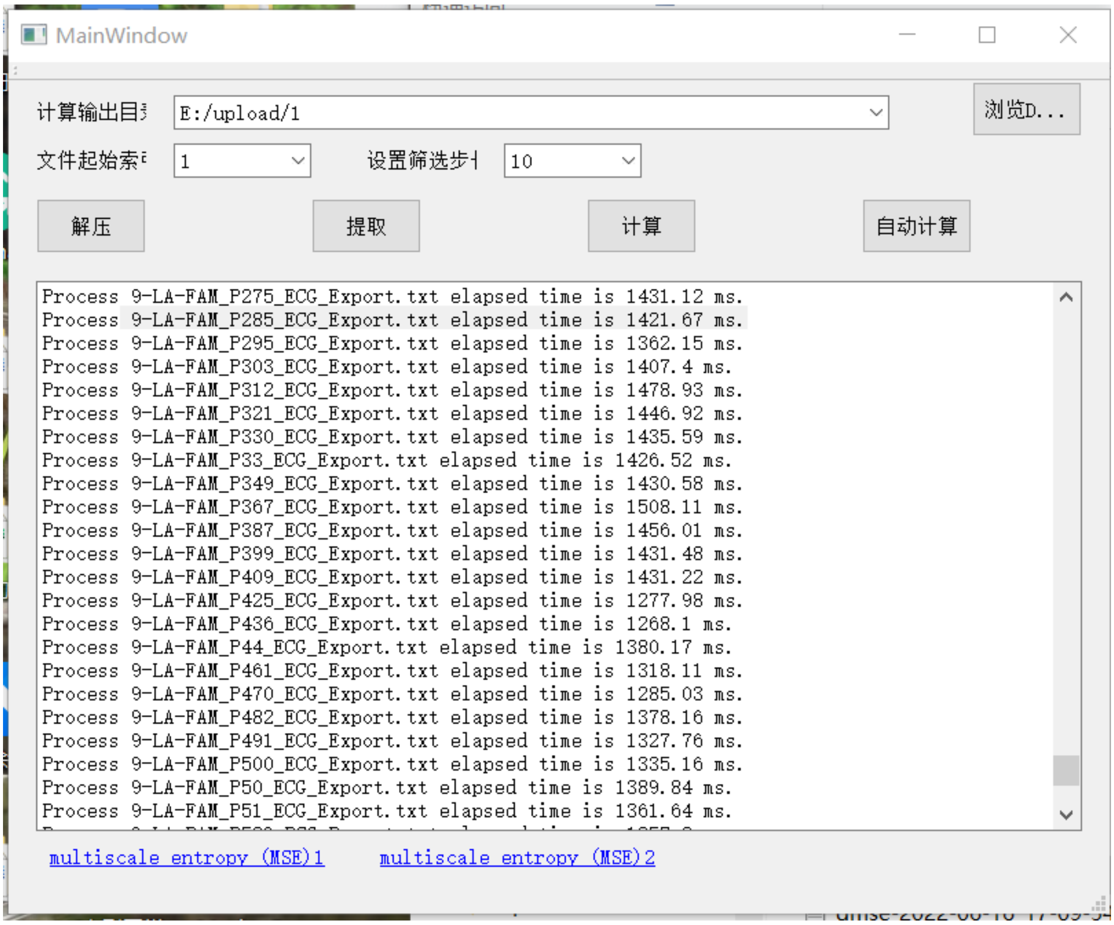
MainWindow
计算输出目
E:/upload/1
浏览D...
文件起始索
1
设置筛选步
10
解压
提取
计算
自动计算
Process 9-LA-FAM_P275_ECG_Export.txt elapsed time is 1431.12 ms.
Process 9-LA-FAM_P285_ECG_Export.txt elapsed time is 1421.67 ms.
Process 9-LA-FAM_P295_ECG_Export.txt elapsed time is 1362.15 ms.
Process 9-LA-FAM_P303_ECG_Export.txt elapsed time is 1407.4 ms.
Process 9-LA-FAM_P312_ECG_Export.txt elapsed time is 1478.93 ms.
Process 9-LA-FAM_P321_ECG_Export.txt elapsed time is 1446.92 ms.
Process 9-LA-FAM_P330_ECG_Export.txt elapsed time is 1435.59 ms.
Process 9-LA-FAM_P33_ECG_Export.txt elapsed time is 1426.52 ms.
Process 9-LA-FAM_P349_ECG_Export.txt elapsed time is 1430.58 ms.
Process 9-LA-FAM_P367_ECG_Export.txt elapsed time is 1508.11 ms.
Process 9-LA-FAM_P387_ECG_Export.txt elapsed time is 1456.01 ms.
Process 9-LA-FAM_P399_ECG_Export.txt elapsed time is 1431.48 ms.
Process 9-LA-FAM_P409_ECG_Export.txt elapsed time is 1431.22 ms.
Process 9-LA-FAM_P425_ECG_Export.txt elapsed time is 1277.98 ms.
Process 9-LA-FAM_P436_ECG_Export.txt elapsed time is 1268.1 ms.
Process 9-LA-FAM_P44_ECG_Export.txt elapsed time is 1380.17 ms.
Process 9-LA-FAM_P461_ECG_Export.txt elapsed time is 1318.11 ms.
Process 9-LA-FAM_P470_ECG_Export.txt elapsed time is 1285.03 ms.
Process 9-LA-FAM_P482_ECG_Export.txt elapsed time is 1378.16 ms.
Process 9-LA-FAM_P491_ECG_Export.txt elapsed time is 1327.76 ms.
Process 9-LA-FAM_P500_ECG_Export.txt elapsed time is 1335.16 ms.
Process 9-LA-FAM_P50_ECG_Export.txt elapsed time is 1389.84 ms.
Process 9-LA-FAM_P51_ECG_Export.txt elapsed time is 1361.64 ms.
multiscale entropy (MSE)1
multiscale entropy (MSE)2

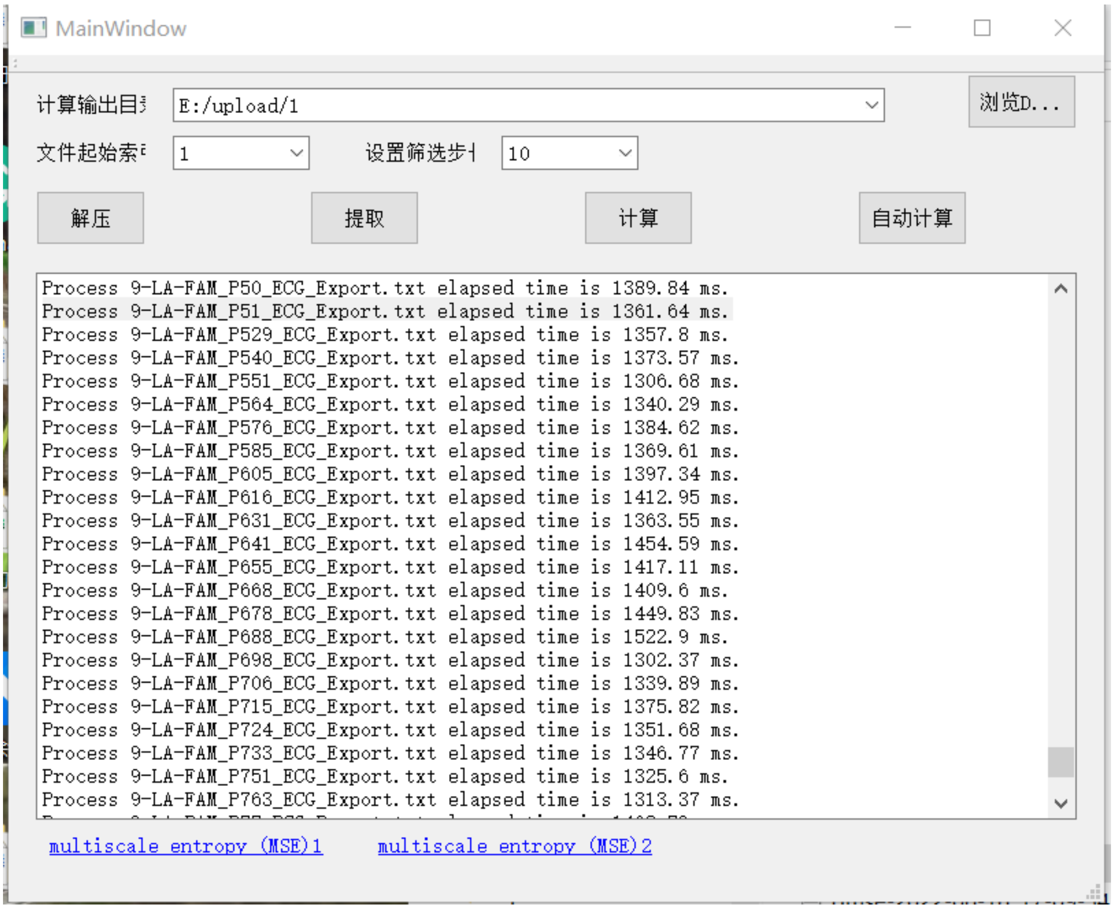
MainWindow
计算输出目
E:/upload/1
浏览D...
文件起始索
1
设置筛选步
10
解压
提取
计算
自动计算
Process 9-LA-FAM_P50_ECG_Export.txt elapsed time is 1389.84 ms.
Process 9-LA-FAM_P51_ECG_Export.txt elapsed time is 1361.64 ms.
Process 9-LA-FAM_P529_ECG_Export.txt elapsed time is 1357.8 ms.
Process 9-LA-FAM_P540_ECG_Export.txt elapsed time is 1373.57 ms.
Process 9-LA-FAM_P551_ECG_Export.txt elapsed time is 1306.68 ms.
Process 9-LA-FAM_P564_ECG_Export.txt elapsed time is 1340.29 ms.
Process 9-LA-FAM_P576_ECG_Export.txt elapsed time is 1384.62 ms.
Process 9-LA-FAM_P585_ECG_Export.txt elapsed time is 1369.61 ms.
Process 9-LA-FAM_P605_ECG_Export.txt elapsed time is 1397.34 ms.
Process 9-LA-FAM_P616_ECG_Export.txt elapsed time is 1412.95 ms.
Process 9-LA-FAM_P631_ECG_Export.txt elapsed time is 1363.55 ms.
Process 9-LA-FAM_P641_ECG_Export.txt elapsed time is 1454.59 ms.
Process 9-LA-FAM_P655_ECG_Export.txt elapsed time is 1417.11 ms.
Process 9-LA-FAM_P668_ECG_Export.txt elapsed time is 1409.6 ms.
Process 9-LA-FAM_P678_ECG_Export.txt elapsed time is 1449.83 ms.
Process 9-LA-FAM_P688_ECG_Export.txt elapsed time is 1522.9 ms.
Process 9-LA-FAM_P698_ECG_Export.txt elapsed time is 1302.37 ms.
Process 9-LA-FAM_P706_ECG_Export.txt elapsed time is 1339.89 ms.
Process 9-LA-FAM_P715_ECG_Export.txt elapsed time is 1375.82 ms.
Process 9-LA-FAM_P724_ECG_Export.txt elapsed time is 1351.68 ms.
Process 9-LA-FAM_P733_ECG_Export.txt elapsed time is 1346.77 ms.
Process 9-LA-FAM_P751_ECG_Export.txt elapsed time is 1325.6 ms.
Process 9-LA-FAM_P763_ECG_Export.txt elapsed time is 1313.37 ms.
multiscale entropy (MSE)1
multiscale entropy (MSE)2

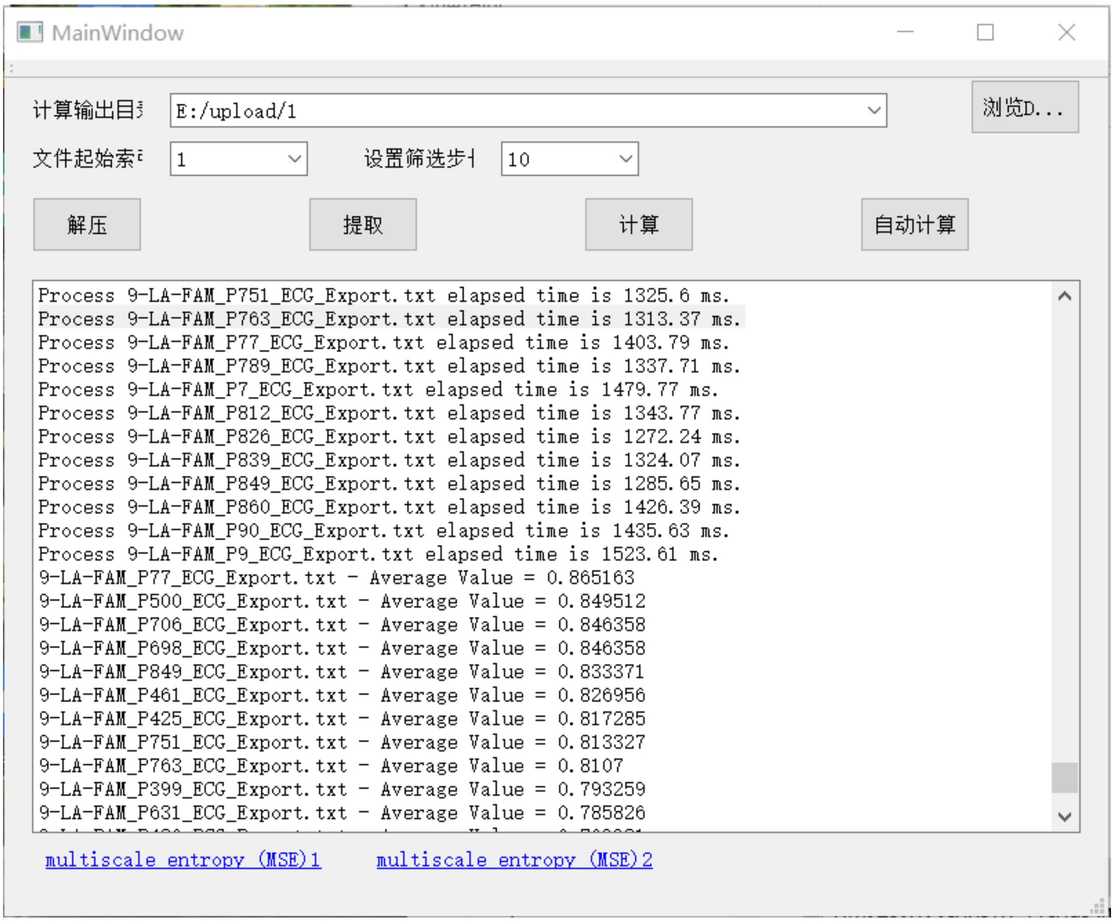
MainWindow
计算输出目
E:/upload/1
浏览D...
文件起始索
1
设置筛选步
10
解压
提取
计算
自动计算
Process 9-LA-FAM_P751_ECG_Export.txt elapsed time is 1325.6 ms.
Process 9-LA-FAM_P763_ECG_Export.txt elapsed time is 1313.37 ms.
Process 9-LA-FAM_P77_ECG_Export.txt elapsed time is 1403.79 ms.
Process 9-LA-FAM_P789_ECG_Export.txt elapsed time is 1337.71 ms.
Process 9-LA-FAM_P7_ECG_Export.txt elapsed time is 1479.77 ms.
Process 9-LA-FAM_P812_ECG_Export.txt elapsed time is 1343.77 ms.
Process 9-LA-FAM_P826_ECG_Export.txt elapsed time is 1272.24 ms.
Process 9-LA-FAM_P839_ECG_Export.txt elapsed time is 1324.07 ms.
Process 9-LA-FAM_P849_ECG_Export.txt elapsed time is 1285.65 ms.
Process 9-LA-FAM_P860_ECG_Export.txt elapsed time is 1426.39 ms.
Process 9-LA-FAM_P90_ECG_Export.txt elapsed time is 1435.63 ms.
Process 9-LA-FAM_P9_ECG_Export.txt elapsed time is 1523.61 ms.
9-LA-FAM_P77_ECG_Export.txt - Average Value = 0.865163
9-LA-FAM_P500_ECG_Export.txt - Average Value = 0.849512
9-LA-FAM_P706_ECG_Export.txt - Average Value = 0.846358
9-LA-FAM_P698_ECG_Export.txt - Average Value = 0.846358
9-LA-FAM_P849_ECG_Export.txt - Average Value = 0.833371
9-LA-FAM_P461_ECG_Export.txt - Average Value = 0.826956
9-LA-FAM_P425_ECG_Export.txt - Average Value = 0.817285
9-LA-FAM_P751_ECG_Export.txt - Average Value = 0.813327
9-LA-FAM_P763_ECG_Export.txt - Average Value = 0.8107
9-LA-FAM_P399_ECG_Export.txt - Average Value = 0.793259
9-LA-FAM_P631_ECG_Export.txt - Average Value = 0.785826
multiscale entropy (MSE)1
multiscale entropy (MSE)2

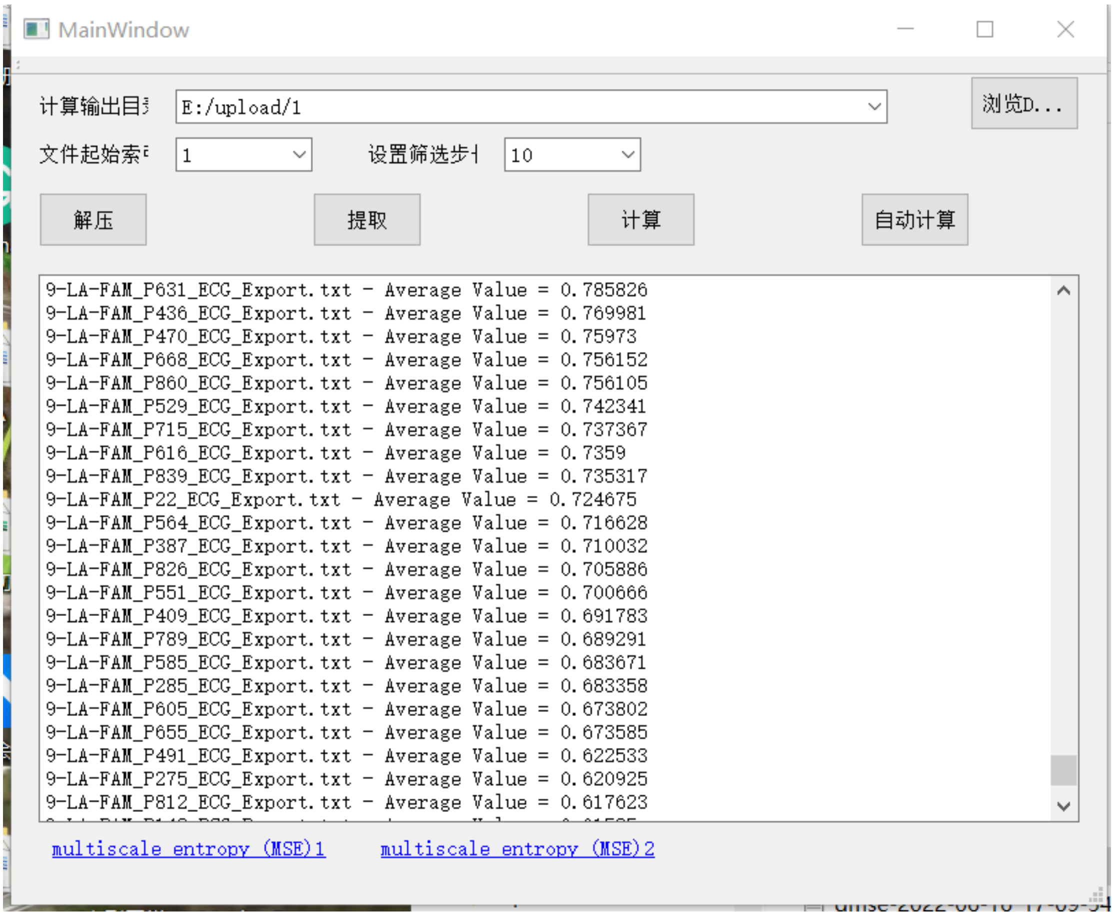
MainWindow
计算输出目
E:/upload/1
浏览D...
文件起始索
1
设置筛选步
10
解压
提取
计算
自动计算
9-LA-FAM_P631_ECG_Export.txt - Average Value = 0.785826
9-LA-FAM_P436_ECG_Export.txt - Average Value = 0.769981
9-LA-FAM_P470_ECG_Export.txt - Average Value = 0.75973
9-LA-FAM_P668_ECG_Export.txt - Average Value = 0.756152
9-LA-FAM_P860_ECG_Export.txt - Average Value = 0.756105
9-LA-FAM_P529_ECG_Export.txt - Average Value = 0.742341
9-LA-FAM_P715_ECG_Export.txt - Average Value = 0.737367
9-LA-FAM_P616_ECG_Export.txt - Average Value = 0.7359
9-LA-FAM_P839_ECG_Export.txt - Average Value = 0.735317
9-LA-FAM_P22_ECG_Export.txt - Average Value = 0.724675
9-LA-FAM_P564_ECG_Export.txt - Average Value = 0.716628
9-LA-FAM_P387_ECG_Export.txt - Average Value = 0.710032
9-LA-FAM_P826_ECG_Export.txt - Average Value = 0.705886
9-LA-FAM_P551_ECG_Export.txt - Average Value = 0.700666
9-LA-FAM_P409_ECG_Export.txt - Average Value = 0.691783
9-LA-FAM_P789_ECG_Export.txt - Average Value = 0.689291
9-LA-FAM_P585_ECG_Export.txt - Average Value = 0.683671
9-LA-FAM_P285_ECG_Export.txt - Average Value = 0.683358
9-LA-FAM_P605_ECG_Export.txt - Average Value = 0.673802
9-LA-FAM_P655_ECG_Export.txt - Average Value = 0.673585
9-LA-FAM_P491_ECG_Export.txt - Average Value = 0.622533
9-LA-FAM_P275_ECG_Export.txt - Average Value = 0.620925
9-LA-FAM_P812_ECG_Export.txt - Average Value = 0.617623
multiscale entropy (MSE)1
multiscale entropy (MSE)2

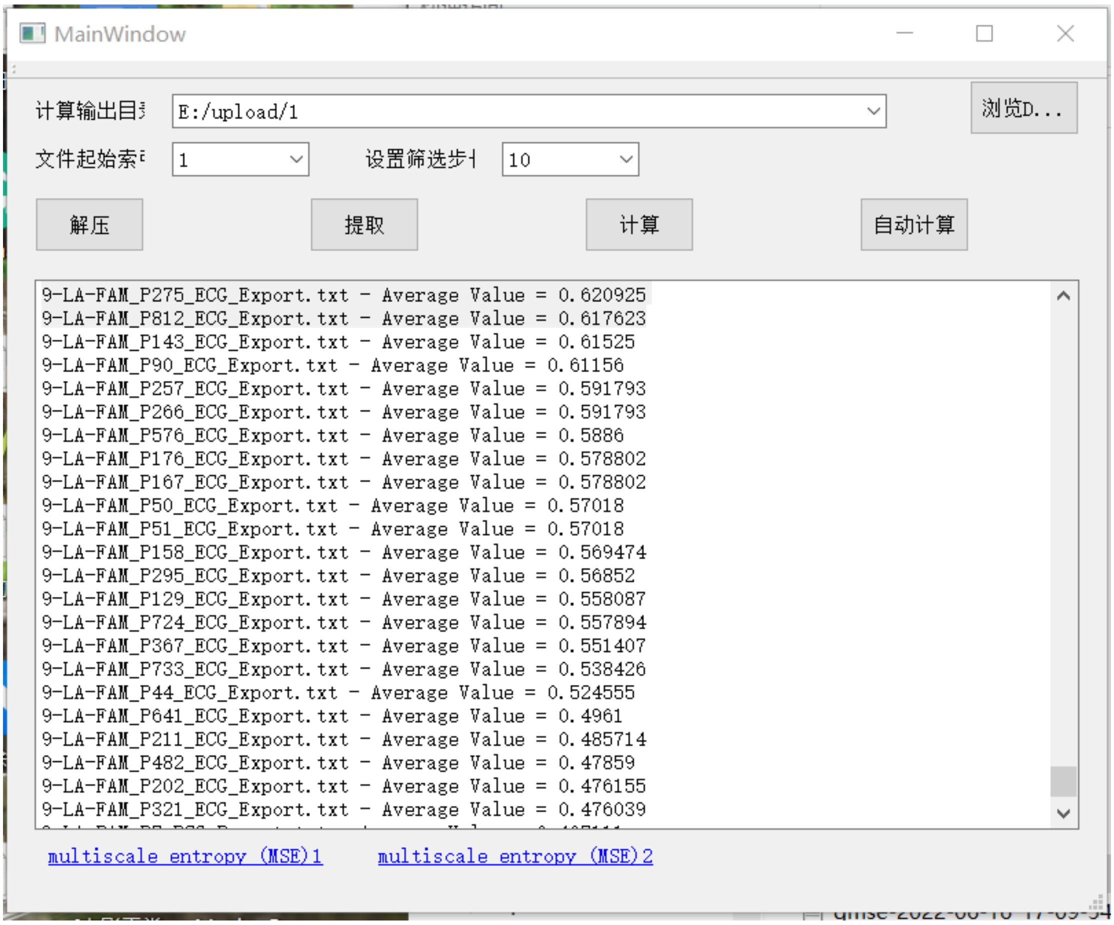
MainWindow
计算输出目录
E:/upload/1
浏览D...
文件起始索引
1
设置筛选步长
10
解压
提取
计算
自动计算
9-LA-FAM_P275_ECG_Export.txt - Average Value = 0.620925
9-LA-FAM_P812_ECG_Export.txt - Average Value = 0.617623
9-LA-FAM_P143_ECG_Export.txt - Average Value = 0.61525
9-LA-FAM_P90_ECG_Export.txt - Average Value = 0.61156
9-LA-FAM_P257_ECG_Export.txt - Average Value = 0.591793
9-LA-FAM_P266_ECG_Export.txt - Average Value = 0.591793
9-LA-FAM_P576_ECG_Export.txt - Average Value = 0.5886
9-LA-FAM_P176_ECG_Export.txt - Average Value = 0.578802
9-LA-FAM_P167_ECG_Export.txt - Average Value = 0.578802
9-LA-FAM_P50_ECG_Export.txt - Average Value = 0.57018
9-LA-FAM_P51_ECG_Export.txt - Average Value = 0.57018
9-LA-FAM_P158_ECG_Export.txt - Average Value = 0.569474
9-LA-FAM_P295_ECG_Export.txt - Average Value = 0.56852
9-LA-FAM_P129_ECG_Export.txt - Average Value = 0.558087
9-LA-FAM_P724_ECG_Export.txt - Average Value = 0.557894
9-LA-FAM_P367_ECG_Export.txt - Average Value = 0.551407
9-LA-FAM_P733_ECG_Export.txt - Average Value = 0.538426
9-LA-FAM_P44_ECG_Export.txt - Average Value = 0.524555
9-LA-FAM_P641_ECG_Export.txt - Average Value = 0.4961
9-LA-FAM_P211_ECG_Export.txt - Average Value = 0.485714
9-LA-FAM_P482_ECG_Export.txt - Average Value = 0.47859
9-LA-FAM_P202_ECG_Export.txt - Average Value = 0.476155
9-LA-FAM_P321_ECG_Export.txt - Average Value = 0.476039
multiscale entropy (MSE)1
multiscale entropy (MSE)2

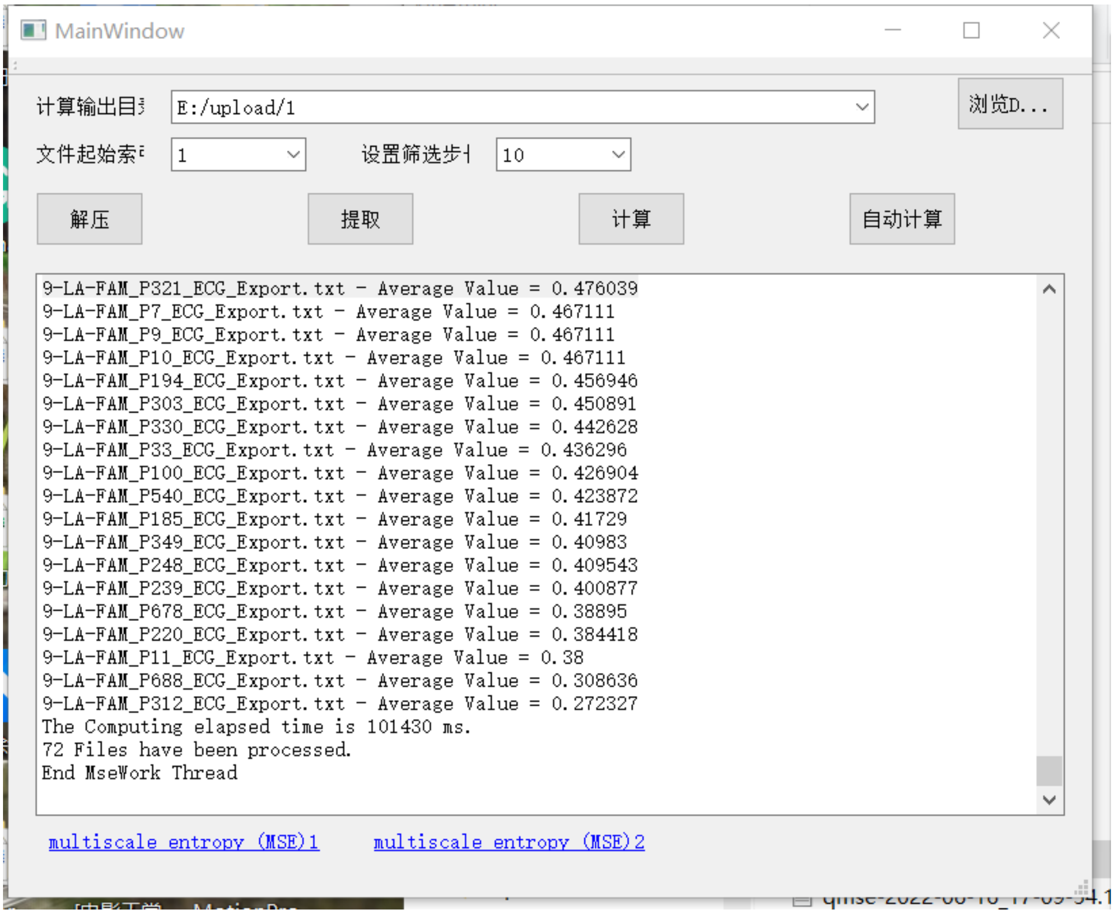
MainWindow
计算输出目录
E:/upload/1
浏览D...
文件起始索引
1
设置筛选步长
10
解压
提取
计算
自动计算
9-LA-FAM_P321_ECG_Export.txt - Average Value = 0.476039
9-LA-FAM_P7_ECG_Export.txt - Average Value = 0.467111
9-LA-FAM_P9_ECG_Export.txt - Average Value = 0.467111
9-LA-FAM_P10_ECG_Export.txt - Average Value = 0.467111
9-LA-FAM_P194_ECG_Export.txt - Average Value = 0.456946
9-LA-FAM_P303_ECG_Export.txt - Average Value = 0.450891
9-LA-FAM_P330_ECG_Export.txt - Average Value = 0.442628
9-LA-FAM_P33_ECG_Export.txt - Average Value = 0.436296
9-LA-FAM_P100_ECG_Export.txt - Average Value = 0.426904
9-LA-FAM_P540_ECG_Export.txt - Average Value = 0.423872
9-LA-FAM_P185_ECG_Export.txt - Average Value = 0.41729
9-LA-FAM_P349_ECG_Export.txt - Average Value = 0.40983
9-LA-FAM_P248_ECG_Export.txt - Average Value = 0.409543
9-LA-FAM_P239_ECG_Export.txt - Average Value = 0.400877
9-LA-FAM_P678_ECG_Export.txt - Average Value = 0.38895
9-LA-FAM_P220_ECG_Export.txt - Average Value = 0.384418
9-LA-FAM_P11_ECG_Export.txt - Average Value = 0.38
9-LA-FAM_P688_ECG_Export.txt - Average Value = 0.308636
9-LA-FAM_P312_ECG_Export.txt - Average Value = 0.272327
The Computing elapsed time is 101430 ms.
72 Files have been processed.
End MseWork Thread
multiscale entropy (MSE)1
multiscale entropy (MSE)2

After that, the top 5 mMSE areas in LA were ablated in a descendent order. The area with the highest mMSE was located at the roof adjacent to the left superior pulmonary vein (green points indicated areas with high mMSE value):

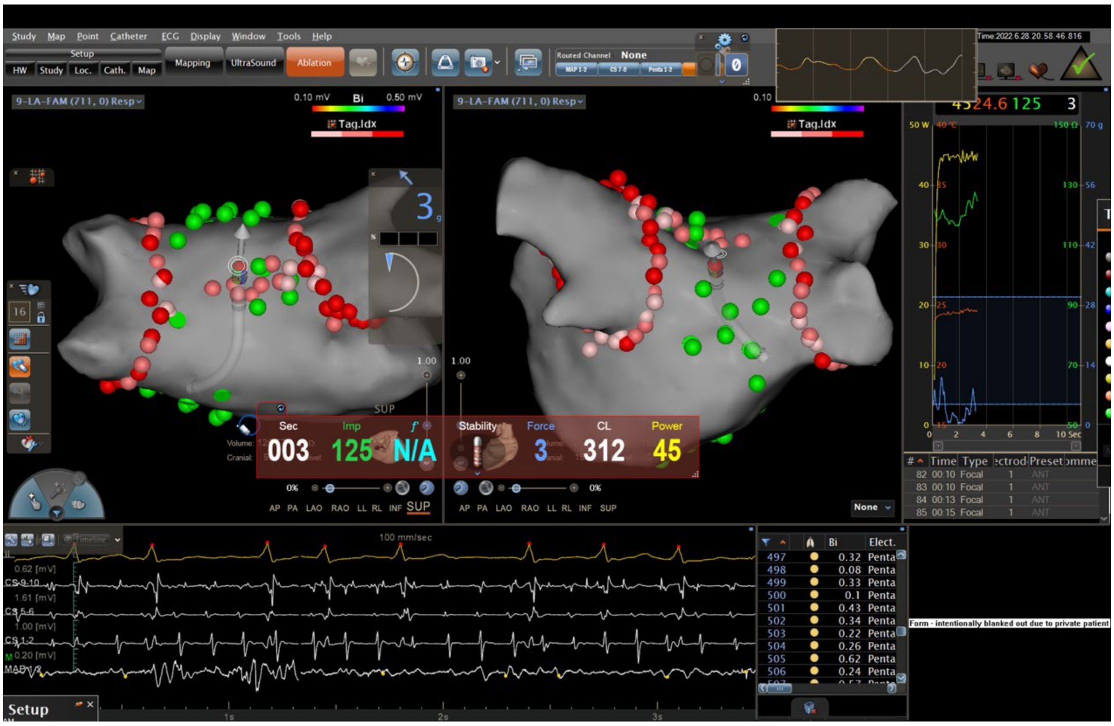

The area with the second highest mMSE was located at posterior wall:

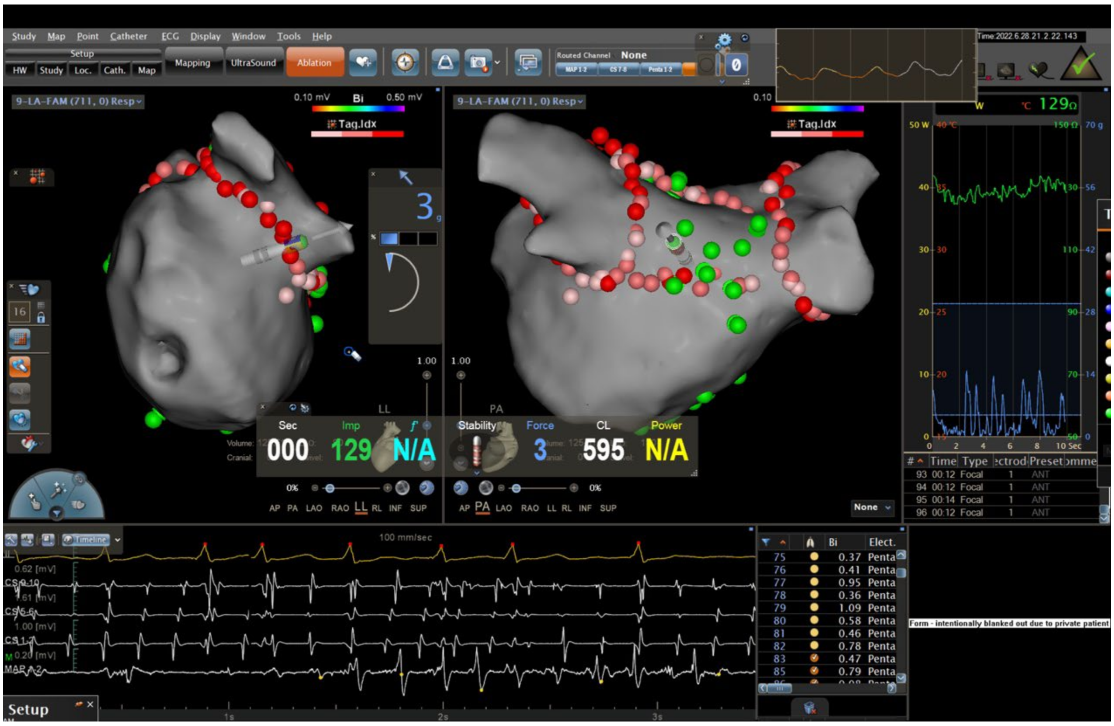

The area with the third highest mMSE was located at the anterior base of right superior pulmonary vein:

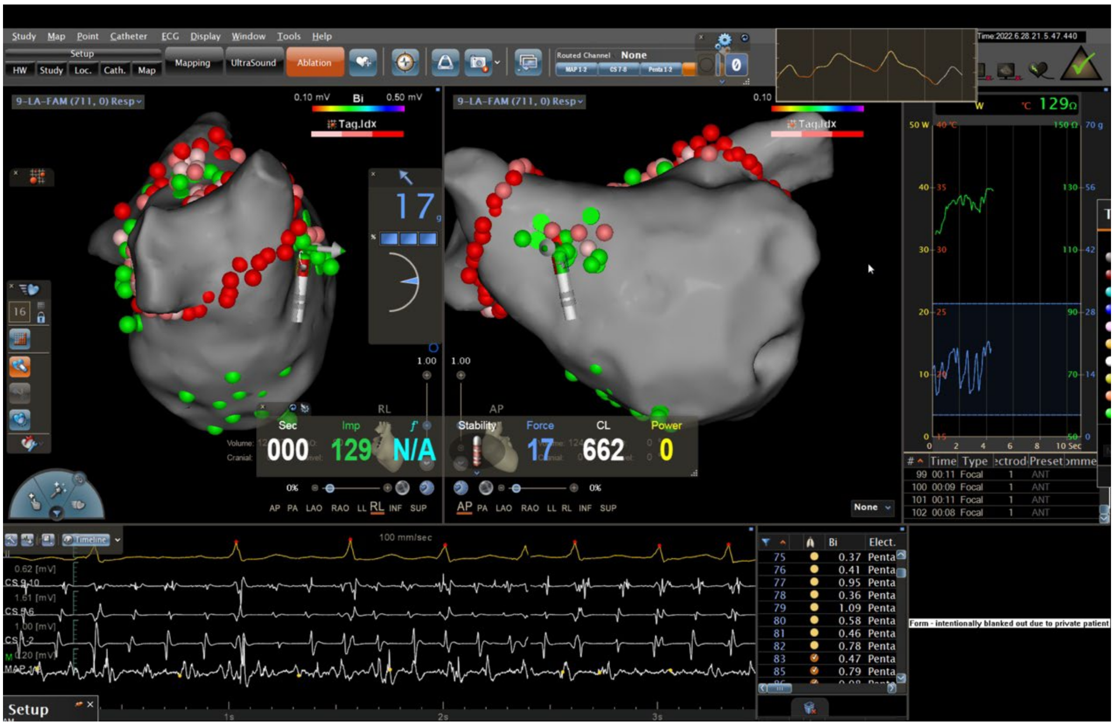

The fourth highest mMSE area was located at the base of the LA:

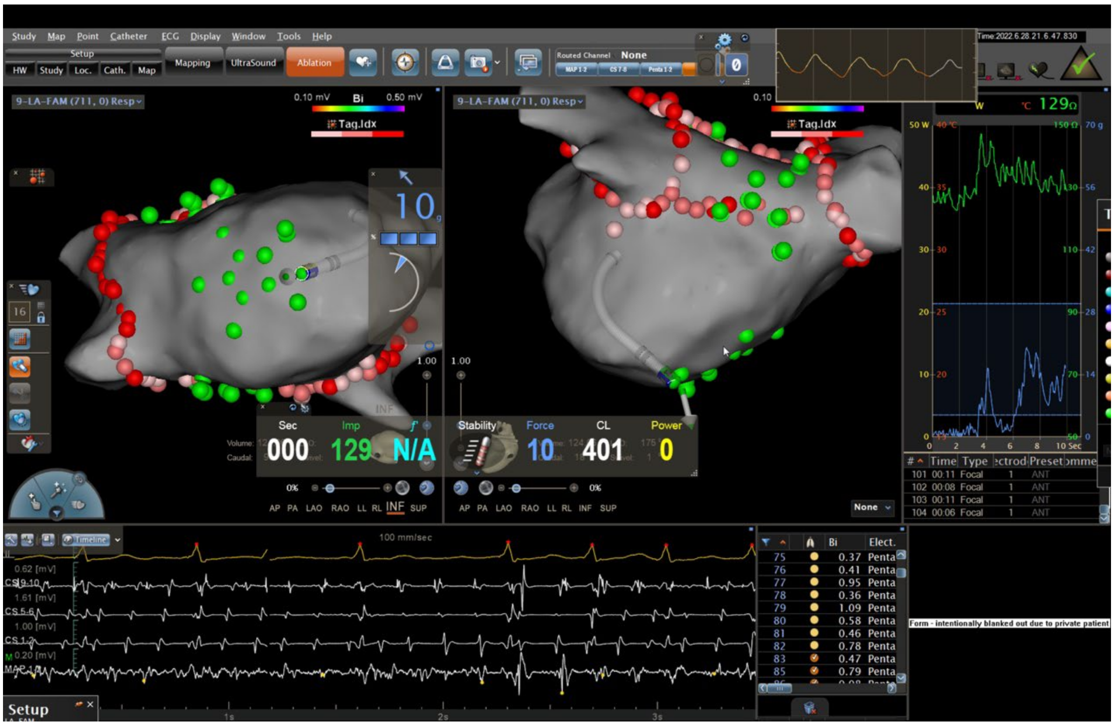

After ablation of this area, AF was terminated, and transformed to atrial flutter:

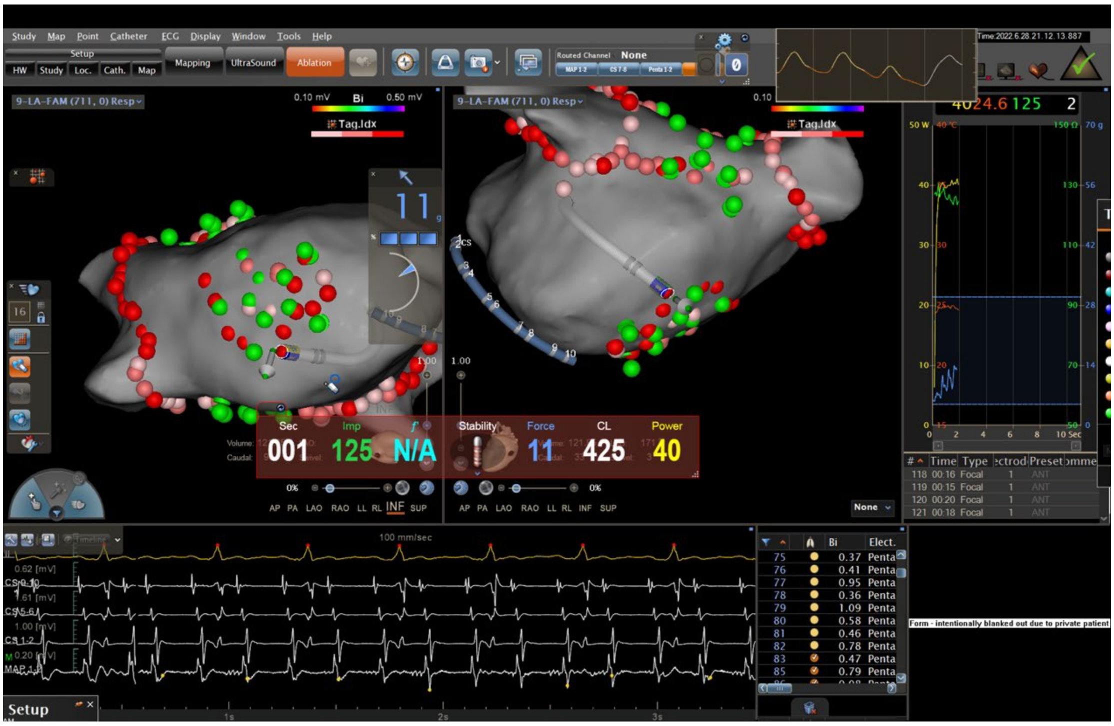

Then the LA was remapped, LAT map showed the atrial flutter was mitral dependent:

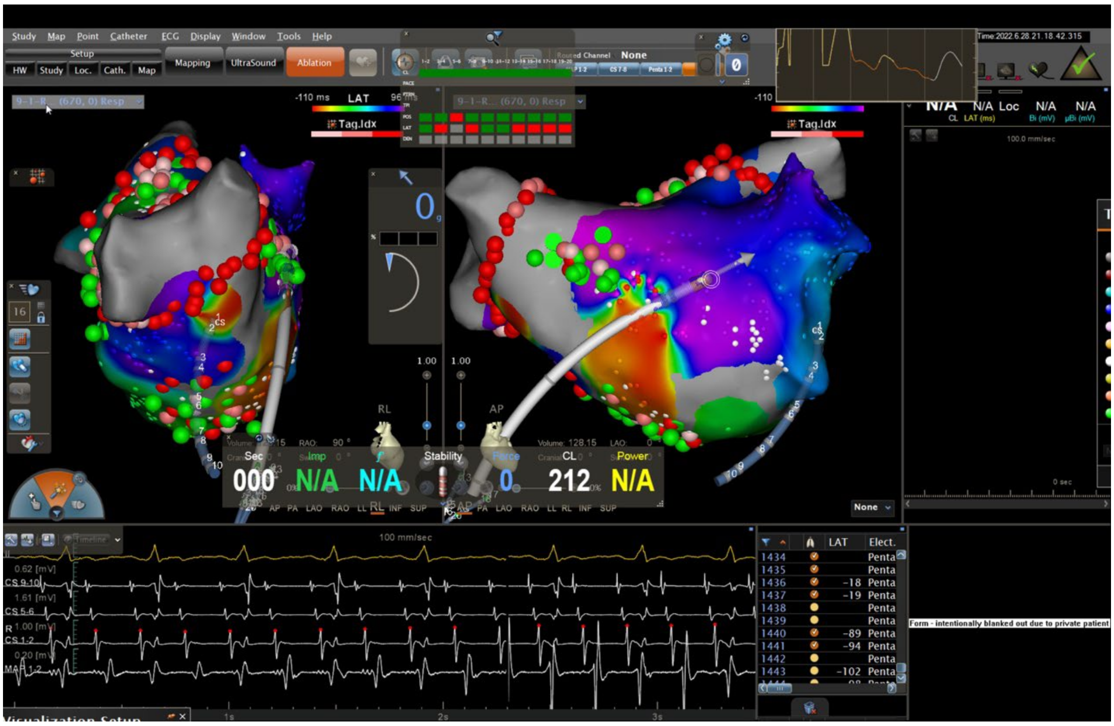

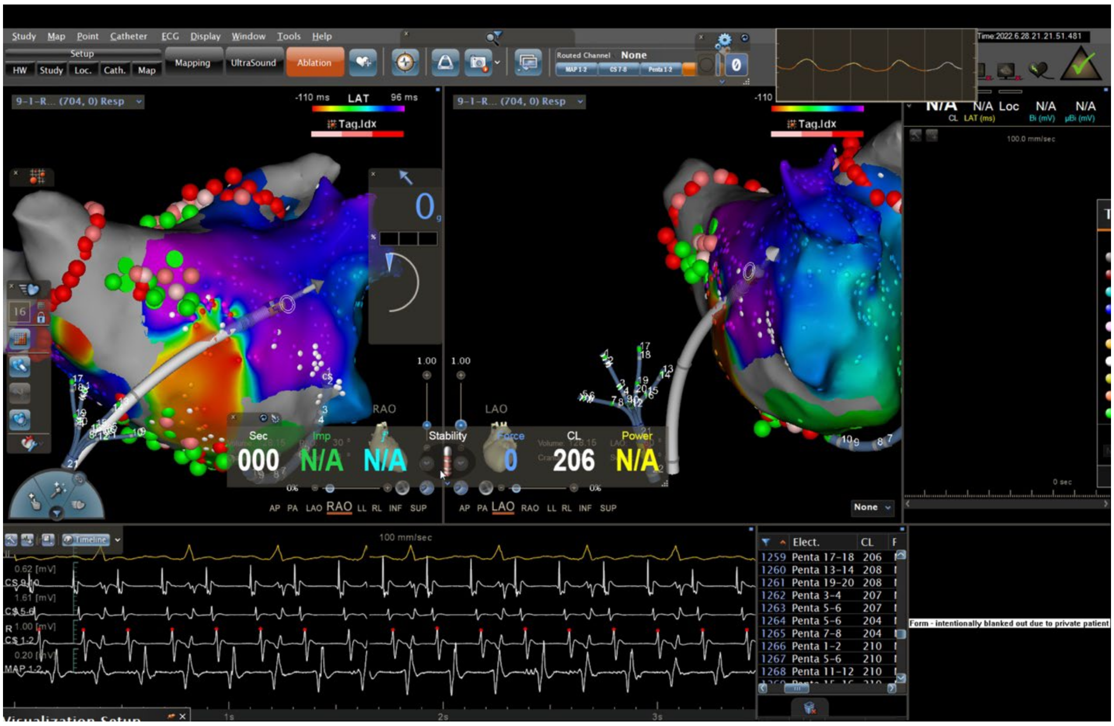

After ablation of mitral line, the flutter changed into tricuspid dependent, then the tricuspid line was ablated, and the sinus rhythm was re-stored during this process:

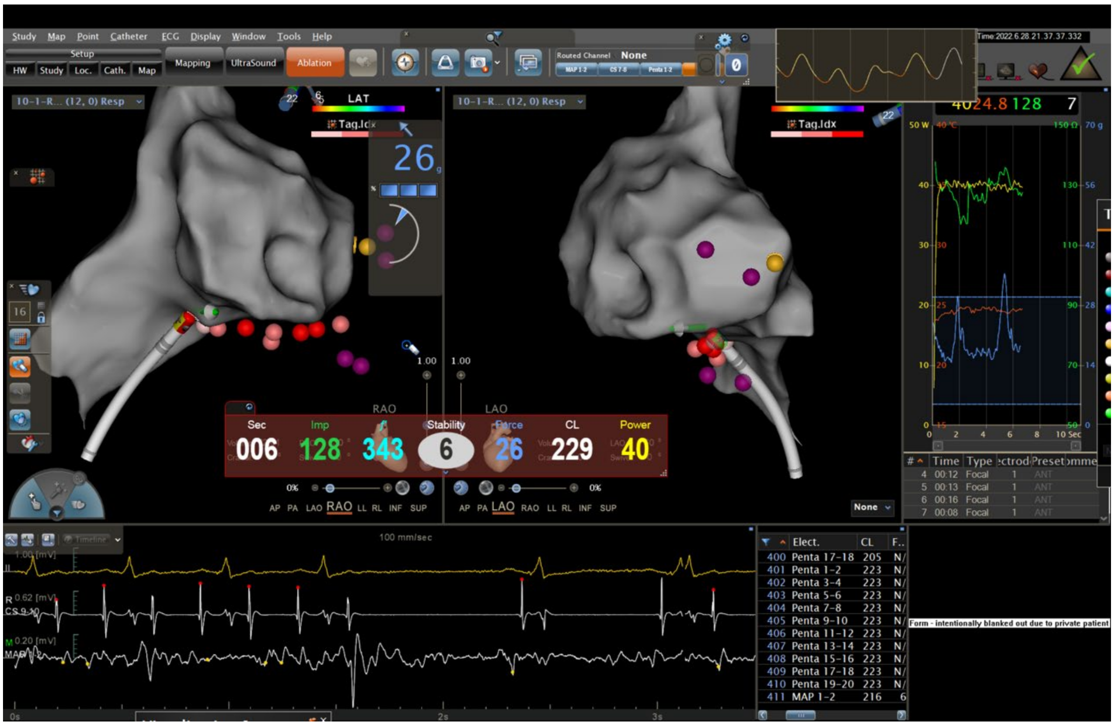

The codes used for mMSE calculation was listed at below:

```
#define _CRT_SECURE_NO_WARNINGS

#include <stdio.h>
#include <string.h>
#include <stdlib.h>
#include <vector>
#include "nmse.h"

using namespace std;

long loadFile(const char* fileName, vector<DATAFV>& dvf, TEXTFV& tfv)
{

	FILE* pFile;
	char sInputBuf[BUFFER_SIZE];
	long lineno = 0L;
	DATAFV dv;


	if ((pFile = fopen(fileName, "rt")) != NULL) {
		while (fgets(sInputBuf, BUFFER_SIZE - 1, pFile) != NULL)
		{
			// skip first line (headers)
			if (++lineno == 1)
			{
				int n = sscanf(sInputBuf, "%s %s %s %s %s %s %s %s %s %s",
					tfv.st[0], tfv.st[1], tfv.st[2], tfv.st[3], tfv.st[4],
					tfv.st[5], tfv.st[6], tfv.st[7], tfv.st[8], tfv.st[9]);
				continue;
			}

			// jump over empty lines
			if (strlen(sInputBuf) == 0)
				continue;

			int n = sscanf(sInputBuf, "%f %f %f %f %f %f %f %f %f %f",
				&dv.fv[0], &dv.fv[1], &dv.fv[2], &dv.fv[3], &dv.fv[4],
				&dv.fv[5], &dv.fv[6], &dv.fv[7], &dv.fv[8], &dv.fv[9]);
			dvf.push_back(dv);
		}

		fclose(pFile);
```

```
	}

	return lineno;
}


void print_datafv(vector<DATAFV>    dvf)
{
	for (vector<DATAFV>::iterator iter = dvf.begin(); iter != dvf.end(); ++iter)
	{
		DATAFV dv = *iter;
		for (int i = 0; i < NUM_FIELDS; i++)
			printf("%f ", dv.fv[i]);

		printf("\n");
	}
 }


int mse_main(const char *filename, const char *outfilename)
{
	FILE* fp;
	int lines = 0L;
	float fv[10];
	int Tao = 20;
	int M = 0;
	int N = 10;
	double* ecg_data = NULL;
	vector<DATAFV>    dvf;
	TEXTFV tfv;
	double MS_Entropy[10][20];

	/*
	char outfilename[256];
	int filelen = strlen(filename);
	int outfilelen = strlen(outpath);

	strncpy(outfilename, outpath,filelen-4);
	strncpy(outfilename, filename,filelen-4);
	strcpy(outfilename+filelen-4,"-result.txt");*/

	lines = loadFile(filename, dvf, tfv);
	//printf("Load File lines = %d\n", lines);
```

```
    M = dvf.size();

    ecg_data = new double[M];


    //% Estimate multi-scale entropy with scal factor = 20
    for (int i = 0; i < N; i++)
    {
        int k = 0;
        for (vector<DATAFV>::iterator iter = dvf.begin(); iter != dvf.end(); ++iter)
        {
            DATAFV dv = *iter;
            ecg_data[k++] = dv.fv[i];
        }

        _nmse(ecg_data, M, MS_Entropy[i], Tao);
    }

    delete ecg_data;


    if ((fp = fopen(outfilename, "wt")) != NULL)
    {
        for (int i = 0; i < 10; i++)
        {
            fprintf(fp, "%s MSE(1--%d): \n", tfv.st[i], Tao);
            for(int j=0;j<Tao;j++)
                fprintf(fp, "%lf\n", MS_Entropy[i][j]);

            fprintf(fp, "\n");
        }

        fclose(fp);
    }

    return(RET_OK);
}
```

```
/**
NMSE.cpp
* Copyright (C) 2022, by Ruize Corp.

% Program for Intracardiac Electrogram (IEGM) Analysis
% The purpose of this program is analyze the raw IEGM data during AF
% using Shannon entropy, Renyi entropy, Multiscale Entropy, Recurrence
% Period Density Entropy, Kurtosis, Empirical Mode Decomposition,
% Dominant Frequency Analysis and Multiscale Frequency.
%
% Subroutine function to calculate Nearest - Neighbor Multi - Scale Entropy
% (NMSE) for the time series data
% data - incoming time series data
% scale - time scale factor chosen to estimate nearest neighbor moving average
% r - Predefined threshold for template matching chosen to be 20 % of the
% standard deviation of the incoming time series data
% MS_E - Returns the multi - scale entropy value of the time series
% This subroutine uses two functions namely NNMA - Nearest - Neighbor Moving
% Average and SampEn which computes the sample entropy of the new time
% series data
**/

#include <math.h>
#include <string.h>
#include "nmse.h"

/*
	求平均值
*/
double _mean(double* data, int datalen)
{
	double sum = 0;
	int N = datalen;

	for (int i = 0; i < N; i++)
	{
		sum += data[i];
	}
	double average = sum / N;
	return average;
}

/*
	计算标准差
```

```
*/
double _std(double* data, int datalen)
{
	int N = datalen;

	double average = _mean(data, N);

	double sum_squares = 0;
	for (int i = 0; i < N; i++)
	{
		sum_squares += (data[i]-average) * (data[i]-average);
	}
	double standard_deviation = sqrt(sum_squares / (N-1));

	return standard_deviation;
}

/**
Subroutine function to calculate NNMA - Nearest - Neighbor Moving
% Averaged time series based on the desired time scale factor 's'
% data - incoming time series data
% s - time scale factor chosen to estimate nearest neighbor moving average
% NNMA - Nearest Neighbor Moving Average - procedure
**/
double NNMA(double* data, int datalen, int s)
{
	int N = datalen; //Estimate the length of the incoming time series data

	// for loop to compute nearest neighbor moving average based on the time
scale factor
	for (int i = 1; i <= N - s; i++)
	{
		if (i > s)	//% check for the presence of backward neighbor
		{
			data[i-1] = _mean(&data[i - s-1], 2 * s + 1); // compute
the average of the corresponding nearest neighbors
		}
	}

	return 0.0;
}

/**
Subroutine function to calculate Nearest-Neighbor Multi-Scale Entropy
```

```
% (NMSE) for the time series data
% data - incoming time series data
% scale - time scale factor chosen to estimate nearest neighbor moving average
% r - Predefined threshold for template matching chosen to be 20% of the
% standard deviation of the incoming time series data
% MS_E - Returns the multi-scale entropy value of the time series
% This subroutine uses two functions namely NNMA- Nearest-Neighbor Moving
% Average and SampEn which computes the sample entropy of the new time
% series data
**/

double _sampEn(double *data, int datalen, double r, int delay)
{
	int N = datalen; // Find the length of the time series
	int	Nn = 0; // Initial value for the number of matched template vectors
for dimension 'm'
	int Nd = 0; // Initial value for the number of matched template vectors for
dimension 'm+1'

	for(int i = 1;i<N - 3 * delay;i++)	// Loop to construct template vectors
in 'm' dimension
		for (int j = i + delay; j <= N - 2 * delay; j++)	// Loop to construct
template vectors in 'm+1' dimension
		{
			if (fabs(data[i-1] - data[j-1]) < r && fabs(data[i + delay-
1] - data[j + delay-1]) < r) // Calculate the Euclidean distance of the template vectors
to be within the threshold 'r' in 'm' dimension
			{
				Nn = Nn + 1;	//increase the # of matched
template vectors by 1 if a matching template vector is found
				if (fabs(data[i + 2 * delay-1] - data[j + 2 *
delay-1]) < r) //Calculate the Euclidean distance of the template vectors to be within
the threshold 'r' in 'm+1' dimension
				{
					Nd = Nd + 1; //increase the # of
matched template vectors by 1 if a matching template vector is found
				}
			}
		}

		double MS_Entropy = -log(Nd*1.0 / Nn);	//Calculate the Sample
entropy as the ratio of matched template vectors in 'm+1' dimension to 'm' dimension

		return MS_Entropy;
```

```
}

void _nmse(double* data, int datalen, double* MS_Entropy, int scale)
{
	double r = 0.2*_std(data, datalen);
	double NT;
	double Entropy;
	double* rdata;

	rdata = new double[datalen];

	for (int i = 1; i <= scale; i++)
	{
		memcpy(rdata, data, datalen * 8);
		NT = NNMA(rdata, datalen, i);
		Entropy = _sampEn(rdata, datalen, r, i);
		MS_Entropy[i - 1] = Entropy;
	}

	delete rdata;

}
```
